# Starch Molecular Structural Features and Volatile Compounds Affecting the Sensory Properties of Polished Australian Wild Rice

**DOI:** 10.3390/foods11040511

**Published:** 2022-02-10

**Authors:** Yingting Zhao, Heather E. Smyth, Keyu Tao, Robert J. Henry, Robert G. Gilbert

**Affiliations:** 1 Jiangsu Key Laboratory of Crop Genetics and Physiology/State Key Laboratory of Hybrid Rice, College of Agriculture, Yangzhou University, Yangzhou 225009, China; yingting.zhao@uqconnect.edu.au (Y.Z.); k.tao@uqconnect.edu.au (K.T.); 2 Jiangsu Key Laboratory of Crop Genomics and Molecular Breeding/Jiangsu Co-Innovation Center for Modern Production Technology of Grain Crops, Yangzhou University, Yangzhou 225009, China; 3 Center for Nutrition and Food Sciences, Queensland Alliance for Agriculture and Food Innovation, The University of Queensland, Brisbane, QLD 4072, Australia; 4 Queensland Alliance for Agriculture and Food Innovation, The University of Queensland, Brisbane, QLD 4072, Australia; h.smyth@uq.edu.au

**Keywords:** rice, descriptive analysis, molecular fine structure, sensory

## Abstract

Cooked high-amylose rices, such as Australian wild rice (AWR) varieties, have slower digestion rates, which is nutritionally advantageous, but may have inferior eating qualities. Here, a comparison is made between sensory and starch molecular fine structure properties, and volatile compounds, of polished AWR varieties and some commercial rices (CRs). Starch structural parameters for amylopectin (Ap) and amylose (Am) were obtained using fluorophore-assisted capillary electrophoresis and size-exclusion chromatography. Volatile compounds were putatively using headspace solid-phase microextraction with gas chromatography-mass spectrometry. Sensory properties were evaluated by a trained panel. AWR had a *disintegration* texture similar to that of Doongara rice, while AWR had a *resinous*, *plastic* aroma different from those of commercial rice varieties. *Disintegration* texture was affected by the amounts of Ap short chains, *resinous* aroma by 2-heptenal, nonadecane, 2h-pyran, tetrahydro-2-(12-pentadecynyloxy)-, and estra-1,3,5(10)-trien-17β-ol, and *plastic* aroma by 2-myristynoyl pantetheine, cis-7-hexadecenoic acid, and estra-1,3,5(10)-trien-17β-ol. These findings suggest that sensory properties and starch structures of AWR varieties support their potential for commercialization.

## 1. Introduction

Rice (*Oryza sativa* L.) is a widely consumed staple food. The health requirement of slow starch digestion rate and the consumer requirement of good palatability of rice are opposing, since cooked high-amylose rices are thus far the only ones with low digestibility, but they have relatively low palatability [1]. Considerable effort has been devoted to finding a rice variety which is both slowly digested and has acceptable sensory properties. One avenue that has been pursued involves Australian wild rice (AWR) varieties, which have significant genetic and, thus, property differences from those of the well-known and widely cultivated *indica* and *japonica* rice varieties [2]. Previous work [3] has shown that AWR starches have more shorter chains of amylose (Am) and more longer chains of amylopectin (Ap), both causing a slower in-vitro digestion rate compared to that of domesticated rices. However, the sensory properties of polished cooked AWR have not been explored. Sensory properties of cooked rice (aroma, appearance, sweet taste, texture, flavor, after-taste, etc.) can be described by panelists (subjective but directly related to human preferences) and by instruments (objective but not directly related to human preferences). There are currently only sensory data for the aroma, texture, and flavor of unpolished cooked AWR varieties [4].

The largest component of the rice endosperm is starch (69–87% on a dry basis). The molecular fine structure of starch is a major controlling influence on textural properties [5], while volatile compounds have a significant effect on rice aroma [6]. Rice flavor results from two broad classes of compounds: those responsible for taste and those responsible for odors [7]. Some sensory properties of unpolished AWR varieties are very different from those of unpolished CRs, such as Long grain, Medium grain, Basmati, Nipponbare, etc. [4]; the understanding of this involves knowing the relationships between starch molecular fine structure, volatile compounds, and sensory properties. Previous studies have found that starch molecular fine structure plays an important role in determining texture, as evaluated by panelists [5,8]. On the other hand, sensory properties, including aroma and flavor, also influence consumer choices [9]. There are only limited studies on how volatile compounds affect the aroma of polished AWR varieties. For example, it has been shown [4] that unpolished AWR has a mild aroma and flavor similar to those of red rice and red basmati, suggesting that AWR would be accepted by consumers. Rice bran has a generally unacceptable flavor to consumers, such as harsh taste [10]; thus, polished AWR is more likely to be accepted by many consumers. It is noted, however, that this depends on cultural preferences: for example, brown (unpolished white) rice is quite acceptable to most consumers in Western countries. There is a paucity of data on the sensory properties of polished cooked AWR varieties, and on correlating their structural parameters with texture.

Our hypothesis is that there are significant differences in molecular fine structure, which play a significant role in determining other level structures, between AWR starches and commercial rice (CR) starches, and there are significant differences in volatile compounds between cooked AWR varieties and CRs, leading to their different sensory properties, and the sensory properties of AWR are acceptable to consumers. If so, AWR will be a rice variety which is both slowly digested (confirmed in our previous work [3]) and has acceptable sensory properties.

The aim of this study is to define the sensory differences between AWR varieties and commercial varieties and to determine if the sensory properties of polished AWR varieties are acceptable to consumers. This study also examined the relationships between starch molecular fine structure, volatile compounds, and sensory properties. This involved the following:

(1) Studying the sensory properties of a polished AWR and CR counterparts.
(2) Characterization of the molecular size distributions of whole branched starch and chain-length distributions (CLDs) of these debranched starches, using size-exclusion chromatography (SEC) for Am and fluorophore-assisted carbohydrate electrophoresis (FACE) for Ap. FACE gives baseline resolution for amylopectin chains but cannot go above a degree of polymerization of 150 and, thus, is essentially confined to amylopectin chains. SEC can be used for any degree of polymerization, but suffers from band-broadening and uncertainties arising from the assumptions needed to convert SEC elution volume to degree of polymerization; with this caveat, it is used here for amylose chains. The Ap and Am CLDs were characterized using biosynthesis-based mathematical models [11,12] to obtain biologically-relevant structural parameters for subsequent use in finding structure-property relations.
(3) Volatile compounds were studied by headspace solid-phase microextraction (HS-SPME) coupled with gas chromatography-mass spectrometry (GC-MS).
(4) Model-fitting parameters and the amounts of volatile compounds were correlated with sensory attributes by Pearson correlation.

The digestibility data of the rice samples here is only to confirm that Australian wild rice had slower digestion rates than those of commercial rice varieties. In addition, the chemical composition and physical traits of the rice grains, and their chromatic properties data here, are to show the differences or similarities between Australian wild rice and commercial rices in these aspects. While these properties could be explored in much greater detail, such exploration is not germane to the aims of the present paper.

## 2. Materials and Methods

### 2.1. Materials

AWR varieties are largely found in remote locations, and as yet have neither been domesticated nor grown under controlled conditions (Henry, 2019). In their natural setting, they are found dispersed among other vegetation, and, very importantly, sample collection often requires permission from the traditional land owners, which involves complex regulatory requirements (Sherman and Henry, 2020). For these reasons, a single AWR from *Oryza meridionalis* was the only AWR for which we could obtain sufficient sample for sensory analysis. This was collected at Global Positioning System (GPS) latitude S 15°49′74.7″ and GPS longitude E: 144°31′39.7″, in Queensland, Australia, in May 2019. The AWR was dehulled manually and then polished with a rice polisher (Model TP-3000 Kett, Tokyo, Japan). Seven CRs (chosen at random), including Sushi, Doongara, Sona Masoori, Ponni Raw, Paella, Long grain, and Australian Medium grain, were purchased from various retailers in Brisbane (Table 1). These rice samples were stored at room temperature in air-tight plastic bags before use.

Protease (from *Streptomyces griseus* type XIV) (P5147), α-amylase from human saliva (A1031), pepsin from porcine gastric mucosa (P6887), and pancreatin from porcine pancreas (P7545) were purchased from Sigma-Aldrich Pty. Ltd. (Castle Hill, Australia). The amyloglucosidase (from *Aspergillus niger*) (3 260 Units/mL) and isoamylase (from *Pseudomonas* sp.) (200 Units/mL) were purchased from Megazyme International Ltd. (Wicklow, Ireland). The 2-methyl-3-heptanone (103128) was purchased from Sigma-Aldrich Pty. Ltd. (Shanghai, China). Pullulan SEC standards with known peak molecular weights ranging from 180 to 1.22 × 10^6^ were obtained from Polymer Standards Service (PSS) GmbH (Mainz, Germany). Other chemical reagents were analytical grade and used as received.

### 2.2. In-Vitro Digestion

The in-vitro digestion method was performed with the method of Zhao et al. [3] with slight modifications. Magnetic stirring bars were added to each 50 mL centrifuge tube. Seventy mg raw whole grains were cooked with 2 mL water for the minimum cooking time (shown in Table 1) for each rice type. This minimum cooking time was found as follows. After boiling the rice (rice:water = 1:4) to 100 °C, measurements were taken after 10 min of cooking and every minute thereafter. The measurements consisted of pressing 10 grains between two glass slides. The time when at least 95% of the 10 boiled grains no longer displayed opaque cores (un-gelatinized centers) was recorded as the minimum cooking time. The oral phase was simulated by passing the cooked rices through a 1.27 mm sieve. Samples were incubated with 0.2 mL artificial-saliva solution of 250 U/mL pancreatic α-amylase in carbonate buffer at pH 7 containing 21.1 mM KCl, 1.59 mM CaCl_2_, and 0.2 mM MgCl_2_ at ambient temperature for 20 s, followed by incubation with porcine pepsin (1 mg/mL) in HCl solution (1 mL, 0.02 M) in a water bath at 37 °C and 100 rpm for 30 min. The digesta were then neutralized with 1 mL NaOH (0.02 M) and mixed with 5 mL sodium acetate buffer (pH 6, 0.2 M) containing 200 mM CaCl_2_, 0.49 mM MgCl_2_, and 0.02% *w*/*v* NaN_3_. Pancreatin (2 mg/mL) and 28 U/mL amyloglucosidase in the same sodium acetate buffer solution (1 mL) were added to the digesta and the mixture incubated in a water bath at 37 °C and 100 rpm. An exactly 0.1 mL aliquot of this mixture was pipetted at certain time intervals (0, 5, 10, 15, 20, 30, 45, 60, 90, 120, 150, and 180 min), and these aliquots were transferred to a microcentrifuge tube containing 0.9 mL absolute ethanol to stop the reaction. The result was centrifuged at 4000× *g* for 10 min. The starch digestibility of cooked rice grains was determined from the amount of glucose released into the supernatant as measured using an enzymatic glucose oxidase reagent TR15104 (Thermo Electron Australia Pty Ltd., Scoresby, VIC, Australia), followed by the measurement of the absorbance at 510 nm using a UV/Vis spectrophotometer (Pharmacia LKB-Ultraspec III, St. Albans, UK).

### 2.3. Composition of Rice Grains

The total starch content was measured using a Megazyme total starch (AA/AMG) assay kit as described elsewhere [3]; crude protein content was measured by a Leco CHNS-928 analyzer (Leco Corp., St. Joseph, MO, USA) (by the combustion method), calculating the nitrogen content with a conversion factor of 5.95.

### 2.4. Color of Rice Grains

Raw and cooked rice grains were analysed in triplicate for color (CIE L c h color space) using a chromameter CR 310 (Konica Minolta, Tokyo, Japan), where L* represents the lightness of luminance component, and parameters a* and b* (from green to red and from blue to yellow, respectively) represent the two chromatic components. Two derived color parameters, hue angle (*h*°) (*h*° = arctan(b*/a*)) and chroma value (C*) (C* = ((a*)^2^ + (b*)^2^))^1/2^), were also used here.

### 2.5. Starch Extraction from Rice Grains

The extraction of starch from the rice grains was carried out following a previously described method [13]. Briefly, rices were ground into flour by a cryo-grinder (MM400, Netsch, Germany, 10 s at a time for 6 times at 20 s^−1^) before being filtered with a 75-µm sieve. The flour was immersed in 0.45% sodium metabisulfite solution (the volume ratio of rice flour to solution was 1:3) at 4 °C for 0.5 h. Proteins were removed by protease (2.5 Units mL^−1^ of protease in tricine buffer (250 mM, pH 7.5)) (37 °C overnight). After centrifuging at 4000× *g* for 10 min, the supernatant was discarded. The treated flour was washed with a sufficient amount of deionized water six times, then twice precipitated in ethanol. Finally, the extracted starch was freeze-dried (SP Scientific, Vir Tis, BTP-9ESOOX, Warminster, PA, USA) at −80 °C for 48 h.

### 2.6. Rice Cooking and Physical Properties

Polished rice kernels (30 g) were placed in 250-mL beakers with a rice-to-water ratio of 1:4. These were covered with aluminium foil, placed in a water bath, and cooked for the minimum cooking time for each rice type at 100 ºC (Table 1) [14]. Cooked rice samples were drained, and sub-samples (5 g) were distributed immediately into plastic cups (30 mL), sealed with a lid, and placed on a tray in a humidified warming oven (70 °C) prior to sensory evaluation.

The lengths and widths of 20 polished rice grains of each sample were measured using a digital Vernier caliper before and after cooking. The colors of cooked rice grains were analyzed by a chromameter CR 310 (Konica Minolta, Tokyo, Japan). The number of broken grains was counted across 100 polished cooked rice grains for each sample.

### 2.7. Sensory Evaluation

Eleven sensory assessors who had been previously tested for sensory acuity and were experienced in sensory descriptive studies were recruited. The accuracy of the sensory data is evaluated in Section 3.3. A texture analyzer, an electronic tongue, or a miniature extrusion cell can also provide texture data. However, sensory scientists always acknowledge that sensory analysis by trained human panelists, as here, is always better (in regard to the real world) than by instrumentation [8]. The latter is used often because it is less expensive. We have here taken the more difficult, but better, human-panelist method. Although the number of panelists is relatively small, these previous tests had shown that the data they produced was reliable.

The ethics committee (University of Queensland Science, Low & Negligible Risk Ethics Sub-Committee) approved this study (Approval Number: 2020000150). All participants in the sensory evaluation part of the study gave written consent before the sensory evaluation and can choose to terminate their participation at any point of the study. Sensory evaluation followed a previous procedure [4] with slight modifications. Briefly, 11 participants (19–57 years old) who had been previously tested for sensory acuity and who were experienced in sensory descriptive studies were recruited. All panelists participated in all training sessions (8 h, 4 sessions), practice (6 h, 1 session), and formal evaluation sessions (6 h, 3 sessions). The training sessions were conducted to help panelists gain familiarity with the samples and develop descriptive sensory terms, definitions, attributes scales, and a tasting protocol. Thirty-three attributes (15 aroma (*aroma intensity*, *sulfurous*, *eggy*, *green vegetable*, *root vegetables*, *sweet caramelized*, *brown bread*, *earthy*, *cereal/porridge*, *fragrant rice*, *resinous*, *plastic*, *raw cookie dough*, *cardboard*, and *chemical* aroma), 6 texture (*fluffiness*, *cohesiveness*, *firmness*, *stickiness*, *chewiness*, and *disintegration* texture), and 12 flavor (*flavor intensity*, *root vegetables*, *sweet caramelized*, *brown bread*, *earthy*, *cereal/porridge*, *fragrant rice*, *resinous*, *plastic*, *cardboard*, *chemical*, and *bitter* flavor)) were selected by consensus, with the definitions being listed below. There are two differences with the cooked rice texture characteristics used here and those used by Meullenet et al. [15]. Firstly, fluffiness and disintegration are characteristics which were not used in Meullenet’s paper. Secondly, the definition of stickiness here was more complete, which defined a sticky glutinous sensation perceived where the sample readily sticks not only itself (shown in Meullenet’s paper) but also the oral surfaces. Toward the end of training, a practice session simulating the formal evaluation was held to confirm the applicability of the method and to evaluate panel performance before formal evaluation. Toward the beginning of the evaluation session, panelists reviewed the attribute definitions. The assessment methods agreed by panelists were as follows: lift the lid and assess the aroma in 1 or 2 sniffs, use the back of a spoon to assess fluffiness and cohesiveness, take ½ teaspoon of sample in the mouth to assess the other texture attributes, and take another half teaspoon of sample in the mouth and assess flavor. Attributes, namely *aroma intensity*, *fluffiness*, *cohesiveness*, *firmness*, *stickiness*, *chewiness*, *disintegration*, and *flavor intensity*, were rated using an unstructured line scale ranging from none (0) to high (100); the other attributes were scored either zero or one because the aroma and flavor attributes disappeared quickly after a sample was taken for sensory evaluation, and there was not enough time to rate them on an unstructured line scale. Fresh water was used as the palate cleanser, and 30 s was the time between characterizing consecutive samples.

### 2.8. Starch Molecular Structural Characterization

Size-exclusion chromatography, SEC, was used to measure the size distribution of whole molecules and the weight CLD of debranched starches (which are linear polymers), as described previously [16]. SEC separates polymer molecules by molecular size, specifically the hydrodynamic radius (*R*_h_). Briefly, native starch was dissolved in DMSO solution with 0.5% (*w*/*w*) LiBr (DMSO/LiBr) at 80 °C before centrifugation, and the supernatant was injected into the SEC column. The SEC weight distributions of branched starch, *w*(log*R*_h_), were obtained using a LC20AD system (Shimadzu Corporation, Kyoto, Japan) equipped with GRAM pre-column, GRAM 30 and 3000 analytical columns (PSS, Mainz, Germany), and an RID-10A refractive index detector (Shimadzu Corporation, Kyoto, Japan). DMSO/LiBr solution was used as the mobile phase with a flow rate of 0.3 mL/min.

For the CLDs, native starch was dissolved in DMSO/LiBr, absolute ethanol was then added to the supernatant, and the resulting precipitate was debranched with isoamylase prior to freeze-drying. The freeze-dried sample was dissolved in DMSO/LiBr, and the resulting supernatant was then injected into the SEC. The SEC weight distributions of debranched starch were obtained using an LC20AD system (Shimadzu Corporation, Kyoto, Japan) equipped with three columns in sequence (PSS, Mainz, Germany): GRAM pre-column, GRAM 100 and GRAM 1000, and a RID-10A refractive index detector (Shimadzu Corporation, Kyoto, Japan). DMSO/LiBr solution was used as the mobile phase with a flow rate of 0.6 mL/min. For linear polymers, such as debranched starch, there is a unique relation between size (*R*_h_) and molecular weight. Elution volume was converted to *R*_h_ through universal calibration using a series of pullulan standards with known molecular weights and the Mark-Houwink relation and *V*_h_ = 4/3 π *R*_h_^3^. The *R*_h_ can be further converted to *X* (where *X* is the degree of polymerization (DP)) using the Mark-Houwink relation again and *M* = 162.2*X* + 18.0 for debranched starch (where *M* is the sample molecular weight, 162.2 is the molecular weight of the anhydroglucose monomeric unit, and 18.0 that of the additional water in the end groups) [17].

FACE was used to measure the number CLD of debranched Ap prepared in the same way as that for SEC analysis, and labeled by 8-aminopyrene-1,3,6,-trisulfonate as described previously [18].

### 2.9. Fitting Ap and Am CLDs to Models

The Ap and Am starch CLDs were each fitted to biosynthesis-based models using publicly available code [11,12]. Both models assume that different regions in the CLDs are mainly, but not exclusively, formed by enzymes belonging to several enzyme sets, which contain various isoforms of starch synthase (SS), starch branching enzymes (SBE), and starch debranching enzymes (DBE). These models enable the CLDs of Ap and of Am to be fitted by rather complex expressions. For each enzyme set *i*, the contribution to the CLD from this set can be computed from the values of two parameters, *β*_Am,*i*_ (where *i* = 1, 2 … denotes the region dominated by this set) and *h*_Am,*i*_ for amylose; and *β*_Ap,*i*_ and *h*_Ap,*i*_ for amylopectin. The numbering of enzyme set *i* uses non-italic Roman numerals: *i* = i, ii, iii, iv, etc. The parameter *β_i_* is the ratio of the activity of SBE to that of SS in set *I*, and *h_i_* is the relative activity of the SS in the enzyme set.

### 2.10. Analysis of Volatile Compounds

The analysis of volatile compounds of cooked rices were performed following a method described elsewhere with slight modification [19]; this analysis used headspace solid-phase microextraction (HS-SPME), followed by Thermo-Trace 1300 gas chromatography-ISQ7000 mass spectrometry (GC-MS). The selection of the HS-SPME conditions used was based on the methods reported and tested by previous researchers [19]. Briefly, cooked polished rices (3 g) were milled and transferred into a 20-mL headspace vial, and then 10 μL of 2-methyl-3-heptanone (50 μg/mL in *n*-hexane) was added as an internal standard solution for semi-quantification. Some volatile compounds volatilize slowly, requiring vibration to accelerate this volatilization and enough extraction time to adsorb volatiles onto a 1 cm 50/30 μm divinylbenzene/carboxen/polydimethylsiloxane (DVB/CAR/PDMS) fiber (Sulpelco, Bellefonte, PA, USA). To be specific, volatile compounds were extracted by first shaking the grains in an oscillating water bath at 60 °C at 250 rpm for 40 min and then adsorbing volatiles onto a 1 cm 50/30 μm DVB/CAR/PDMS fiber at 60 °C for 50 min. They were subsequently desorbed in the GC injection port for 5 min at 250 °C. Before extraction, the fiber was preconditioned at 250 °C for 30 min. Blanks were not performed between samples.

GC-MS analysis was performed on a Thermo-Trace 1300 gas chromatograph with ISQ 7000 mass spectrometer system using a DB-Wax capillary column (30 m × 0.25 mm × 0.25 μm) for chromatographic separation. Highly purified helium (99.999%) was selected as carrier gas with a flow rate of 1.0 mL/min. The temperature program was set at 40 °C for 3 min, then increased to 150 °C at a rate of 3 °C/min and maintained at 150 °C for 1 min, and then further increased to 230 °C at a rate of 4 °C/min and maintained at 230 °C for 10 min. The ion source temperature and transfer line temperature were both 230 °C. The mass selective detector was operated in an electron impact ionization mode at 70 eV, in a scan range of 20–550 *m*/*z*. All experiments were performed in triplicate. Standards and retention index were not used here, but similarity testing (Supplementary Table S1) based on NIST2017 was done in this study. This is consistent with previous literature [20].

### 2.11. Statistical Analysis

Principal Component Analysis (PCA) was performed to determine the relations between the descriptive attributes and sample grouping by XLSTAT (v2020.5.1, Addinsoft 1995–2021, Paris, France. Analysis of Variance (ANOVA) was used to determine differences in starch characteristics. Pearson correlation was applied to build the relations between starch molecular fine structure and sensory analysis using SPSS software (v27.0, SPSS Inc., Chicago, IL, USA).

## 3. Results and Discussion

### 3.1. Comparison of In-Vitro Digestibility Properties of AWR and CRs

Using two digestion fitting models (the parallel-fitting model [21] and the sequential-fitting model [22]) (Supplementary Figure S1), the digestion rate coefficients of the AWR using both models were found to be lower than those of the CRs (Supplementary Figure S2). This was consistent with our previous paper, where the k values of the AWR varieties were also lower than those of the domesticated rice varieties [3].

### 3.2. Chemical Composition and Physical Traits of Rice Grains

The rice chemical compositions of AWR and CRs are presented in Table 1. No large differences were seen between polished AWR and CR grains in total starch, protein, nor amylose content (AC). The total starch content ranged from 75.9% to 79.4%, the protein content from 10.0% to 13.2%, and the AC from 16.1% to 25.5%.

The dimensions and colors of both raw and cooked AWR and CR grains, and percentage of broken AWR and CR grains, are shown in Table 2, and the visual pictures of these raw rices are shown in Supplementary Figure S3. The length and width of raw rices varied from 4.7 to 7.3 mm and 1.6 to 2.8 mm, respectively, and from 7.6 to 10.4 mm and 2.2 to 3.3 mm, respectively, when considering the cooked rices. The AWR was mid-range in terms of the ratio of length to width compared to the other rice samples. However, no distinct differences could be found to distinguish AWR and CRs in dimensions. With regard to color parameters, the AWR had lower L* (darker) and *h*° (lower hue angle) but higher a*, b*, and C* in both raw and cooked polished rice compared to those of CRs. AWR varieties had positive values of a* (toward the red), while CRs had negative value of a* (toward green). Typically, the AWR samples had the highest percentage of broken grains after cooking (45% broken grains), while Sona Masoori rices were the most intact rices after cooking (1% broken grains).

### 3.3. Sensory Attributes and Evaluation of the Accuracy of the Sensory Data

A list of sensory attributes was developed by a trained sensory panel using conventional descriptive analysis techniques (Table 3). There were 15 aroma, 6 texture, and 12 flavor attributes, selected by consensus. Scales and anchors (0–100) were developed for attributes, namely *aroma intensity*, *fluffiness*, *cohesiveness*, *firmness*, *stickiness*, *chewiness*, *disintegration*, and *flavor intensity*; anchors (0 or 1) were applied to the other attributes, which were so ephemeral that it was hard to rate them in an unstructured line scale. Among these texture attributes, cohesiveness, firmness, and stickiness can also be satisfactorily obtained using a miniature extrusion cell and then analyzed by Spectral Stress Strain Analysis [15]. A team of 11 assessors evaluated each rice sample in triplicate under controlled conditions.

A summary of the F ratios and levels of significance obtained from the mixed-model ANOVA with one fixed effect (sample) and five random effects (panelist, replicate, sample by panelist, sample by replicate and panelist by replicate) is shown in Table 3. The scoring of each attribute was significantly different (*p* < 0.01) across the 8 cooked polished rice samples, meaning the samples were different for all sensory attributes. Additionally, the interaction of sample × panelist was significantly different (*p* < 0.01) across all of the sensory attributes, with the exception of *chewiness*. This indicates that the panel had the ability to distinguish differences among samples for all attributes. There were no differences in the ratings of samples between replicates for all attributes, except for *chewiness* and *disintegration*, indicating that only slight differences existed in replicates. The interaction of panelist × replicate did not differ significantly across attributes, indicating that panelists were consistently rating those attributes across replicates. This implies that the panel performance was robust and was suitable for proceeding with further analysis.

### 3.4. Comparison of Sensory Properties between Cooked AWR and CRs

**Figure 1:**
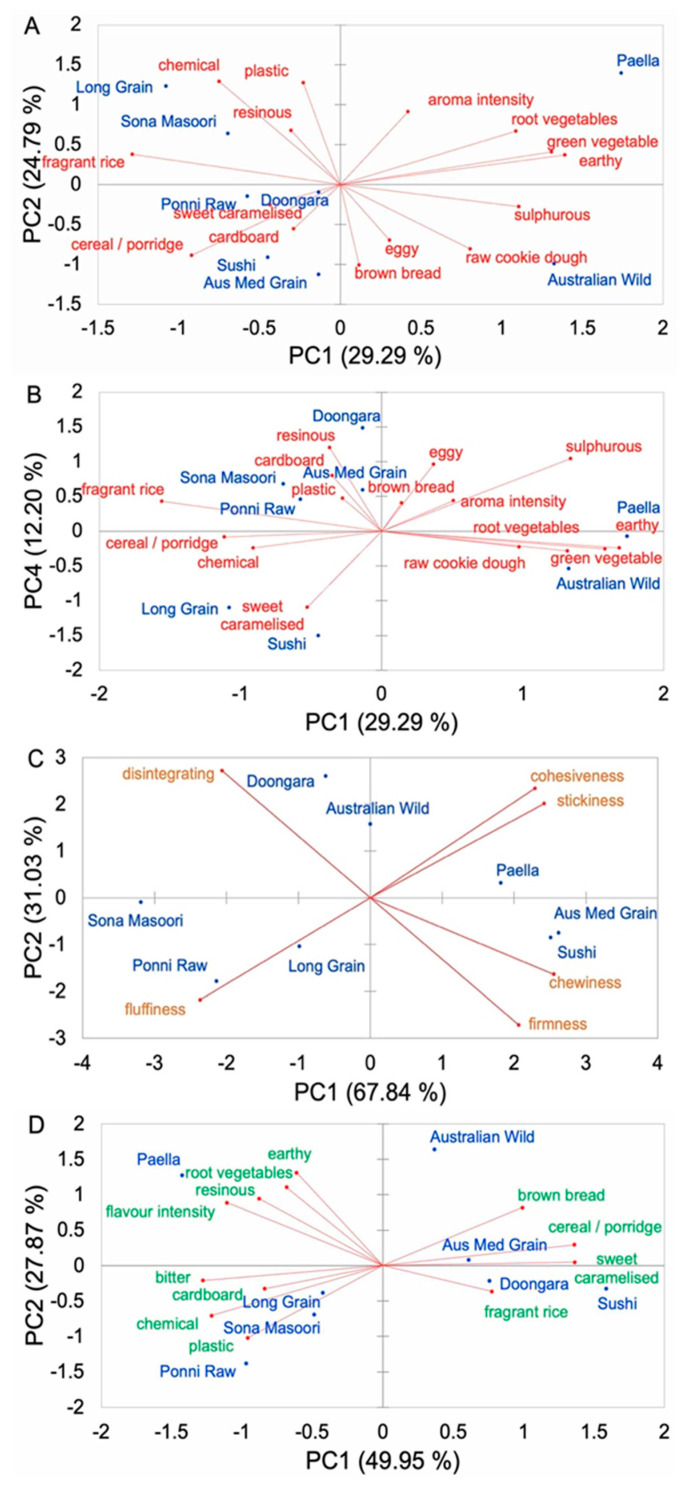
PCA bi-plot of the sensory properties of 8 cooked polished rice samples (*n* = 3 replicates × 11 panelists). (**A**,**B**) aroma attributes; (**C**) texture attributes; (**D**) flavor attributes.

Different PCA bi-plots were used to explore each type of sensory property of the cooked polished rice samples. Generally, the AWR and CRs were distinctly separate in terms of aroma, texture, and flavor, but AWR had some sensory properties similar to those of CR counterparts. For aroma, the AWR was rather distinctive. Attributes, such as *aroma intensity*, *root vegetables*, *green vegetables*, *earthy*, *sulfurous*, *raw cookie dough*, *eggy*, and *brown bread*, are located on the positive side of PC1, while attributes, such as *plastic*, *chemical*, *resinous*, *fragrant rice*, *cereal/porridge*, *sweet caramelized*, and *cardboard*, are located in opposite positions (Figure 1A). The AWR was scored high for *brown bread*, *raw cookie dough*, *sulfurous*, and *eggy*. The AWR was also scored high for *raw cookie dough*, *earthy*, *green vegetable*, and *root vegetables* (Figure 1B). Similarly, Paella rice was also scored higher for *raw cookie dough*, *root vegetables*, *green vegetable*, and *earthy*. The AWR had significant aroma differences from those of CRs, mainly because of *fragrant rice*, *resinous*, and *plastic* aroma. The latter two aromas did not belong to higher eating qualities; therefore, the AWR with lower values of these two aromas tended to have acceptable sensory. Generally, the aroma of the AWR was complex, such as that of *raw cookie dough*, *cereal*, with *brown bread* notes, and some *sulfurous*, *eggy*, *earthy*, and *root vegetable* notes. For texture, the AWR was somewhat different to commercial rice. Samples were differentiated across PC1 by those that were scored high for *cohesiveness*, *stickiness*, *chewiness*, and *firmness* on the positive side of the plot and those that were scored high for *disintegration* and *fluffiness* on the negative side of the plot (Figure 1C). The AWR was scored high for *disintegration*, *cohesiveness*, and *stickiness*, but low for *fluffiness*, *chewiness*, and *firmness*, and these attributes aligned closely with PC2, which means AWR was cohesive and sticky, neither fluffy nor very chewy, and soft and acceptable disintegration in the mouth. Interestingly, the AWR had a *disintegration* texture similar to that of Doongara rice. In addition, the AWR had acceptable *hardness* and *stickiness*, which means that the AWR is preferred by panelists (at least for the cohort used in Reference [5]). For flavor, the AWR was rather distinctive. Flavor differentiated samples across PC1 (Figure 1D) scored higher for *brown bread*, *cereal/porridge*, *sweet caramelized*, and *fragrant rice* on the positive of the plot from those being scored higher for *earthy*, *root vegetables*, *resinous*, *flavor intensity*, *bitter*, *cardboard*, *chemical*, and *plastic* on the negative of the plot. The AWR had significant flavor differences from those of CRs, mainly due to *bitter*, *cardboard*, *chemical*, and *plastic* flavors. These four flavors did not belong in the category of higher eating quality, so AWR varieties with lower values of these flavors tended to have acceptable sensory. The AWR had a *brown bread* flavor slightly similar to that of Australian Medium grain. The AWR was scored high for *cereal/porridge*, *brown bread*, and *sweet caramelized*, and these attributes aligned closely with PC1. The AWR also scored high for *earthy* and *root vegetables* flavors, and these attributes aligned closely with PC2. Paella rice also had these attributes. Generally, the flavor of the AWR was complex, such as *cereal/porridge*, with strong *earthy* and *brown bread* flavors.

### 3.5. Starch Molecular Structure Analysis

**Figure 2:**
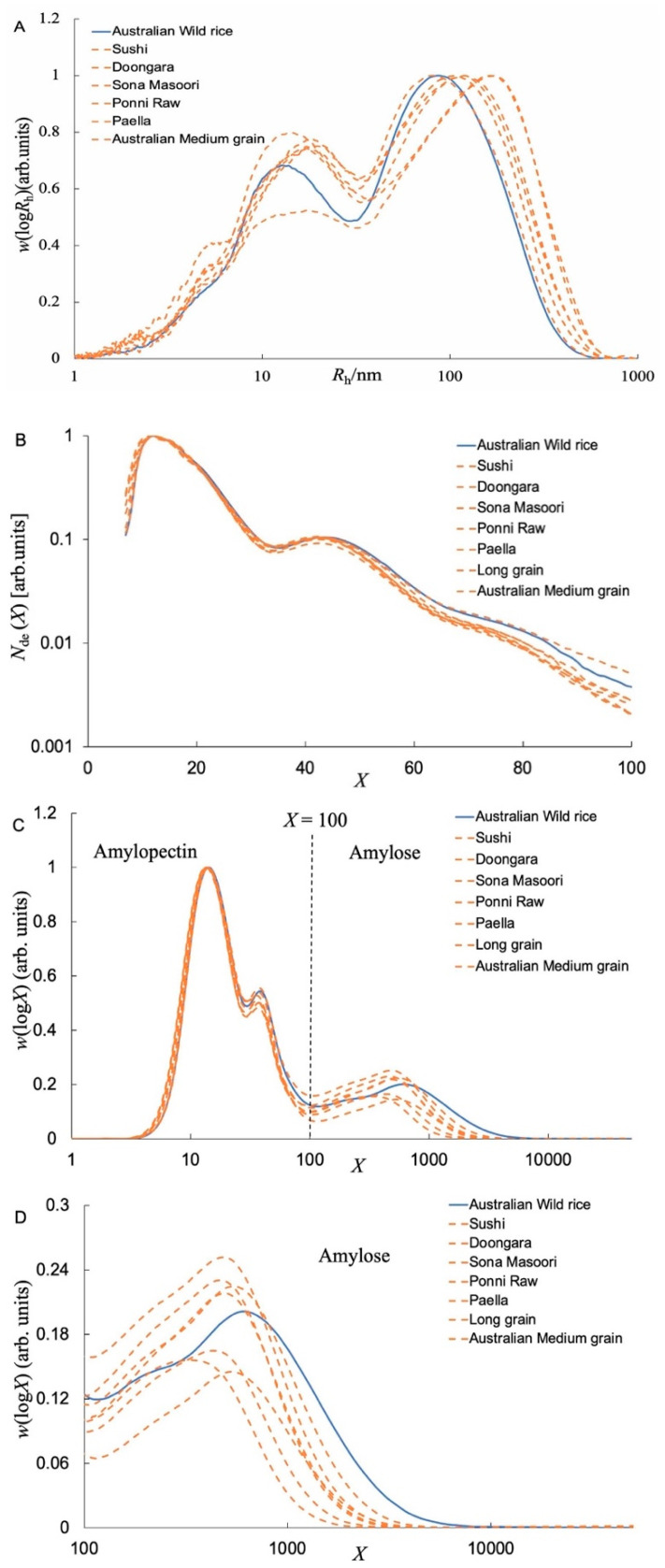
(**A**) SEC weight distributions, *w*(log*R*_h_), of whole (branched) starches extracted from both AWR and CRs as a function of molecular size *R*_h_, normalized to the maximum of Ap component. (**B**) The starch CLDs characterized by FACE plotted as *N*_de_(*X*) against DP *X* (FACE produces discrete points for each DP, but, for visual ease, these are plotted as continuous lines.). (**C**) The starch CLDs characterized by SEC plotted as *w*(log*X*) against DP *X*, normalized to the maximum of the Ap component. (**D**) Enlargements of the Am regions of the CLDs. All data are averages of duplicate measurements.

Figure 2A shows the SEC weight distributions, *w*(log*R*_h_), of whole branched starches, normalized to the peak maximum of Ap. As usual [17], one sees a high Ap peak (*R*_h_ ~ 33 nm) and low peak of Am (6 ≲ *R*_h_ ≲ 33 nm) peaks with a small protein residue shoulder at *R*_h_ over 3 to 6 nm. Although proteins in rice endosperm, especially those combined with starch granules, cannot be fully hydrolyzed by protease [23], small amounts of protein residues were considered to have no impact on SEC characterization due to the different elution volumes [24]. As shown in Table 4, the $\overset{—}{R}$_h,AM_ (the average *R*_h_ of the Am) of AWR was significantly lower than those of CRs. It is noted that larger Ap molecules are more susceptible to shear scission during passage through the SEC than Am because of their large molecular size and relatively inflexible structure [17], but *R*_h_ values can still be compared semi-quantitatively for runs done on the same system with the same settings.

As commonly seen, one has Ap chains over DP ≲ 100 and Am chains for DP ≳ 100. Figure 2B shows the FACE number distributions of debranched Ap extracted from rice. Generally, there are four peaks and/or shoulders for all rice samples, as also seen for CRs.

Figure 2C shows the SEC weight distributions of debranched starch extracted from rice. As usual, there are two peaks for Ap, corresponding to starch chains confined to one crystalline lamella in the native grain and starch chains spanning at least two lamellae, respectively, and then two peaks for Am. An enlargement of Am regions is shown in Figure 2D. The AWR starch has a distinct low-*X* component and a higher maximum *X* compared to those of CR starches. Specifically, the DP of the peak maximum in the amylose range of the AWR was ~610 compared with 326–531 for CR starches. Note that absolute DP values from SEC are only semi-quantitative because of uncertainties in the application of the Mark-Houwink relation and in the values of the Mark-Houwink parameters, but the relative DP values are quite reliable. The AWR starches had shorter short Am chains and longer long Am chains compared to those of CRs.

### 3.6. Comparison of Starch Molecular Structural Parameters of AWR and CRs

**Figure 3:**
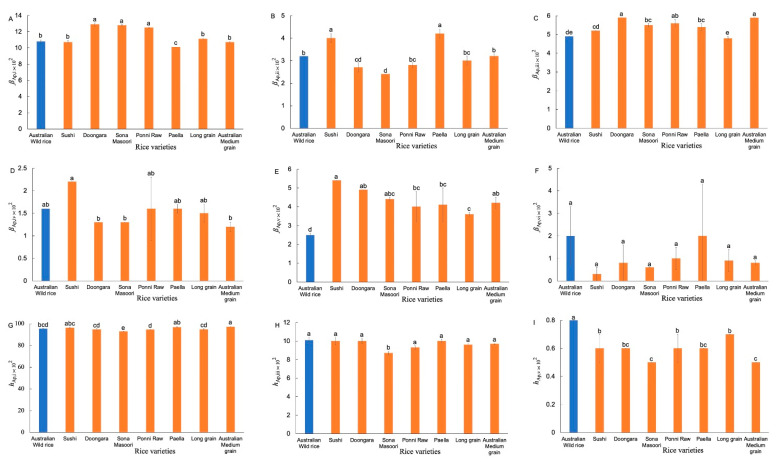
Comparison of Ap structural parameters of AWR starch and CR starches (**A**–**I**). Blue and red: Australian wild and commercial rices, respectively. All data were from duplicate measurements. The same letters mean no significant difference (*p* < 0.05).

The number CLDs of Ap chains (Supplementary Figure S4a) were fitted with an amylopectin biosynthesis-based model [11]. Figure 3 shows the resulting Ap structural parameters. Significant differences were observed in *β*_Ap,v_ and *h*_Ap,v_ values between the AWR starches and commercial ones. The AWR starch had lower *β*_Ap,v_, but higher *h*_Ap,v_, values than that of commercial ones, which means the AWR starch had a higher amount of longer Ap chains (68 < *X* < 97).

**Figure 4:**
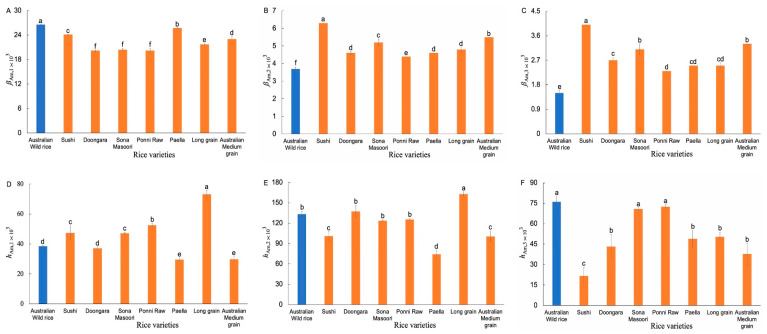
Comparison of Am structural parameters of AWR starch and CR starches (**A**–**F**). Blue and red: Australian wild and commercial rices, respectively. All data were from duplicate measurements. The same letters mean no significant difference (*p* < 0.05).

The weight CLDs of Am chains (Supplementary Figure S4b) were fitted with an amylose biosynthesis-based model [12]. Figure 4 shows the resulting Am structural parameters. Significant differences were observed in *β*_Am,1_, *β*_Am,2_, and *β*_Am,3_ values between the AWR starch and commercial ones. The AWR starch had higher *β*_Am,1_, but lower *β*_Am,2_ and *β*_Am,3_, values than that of commercial ones, which means the AWR starch had shorter Am short chains, as well as longer Am medium-long chains, compared to the CR varieties. 

### 3.7. Determination of Volatile Compounds

A total of 62 volatile compounds were identified in cooked rices (Supplementary Table S1). All volatile compounds have been checked based on similarity and previous literature on cooked rice volatile compounds. The aroma description and odor threshold of these cooked rices was cited from previous literature [25,26,27,28,29,30,31,32,33,34,35,36,37,38,39]. These included 4 acids, 13 alcohols, 9 aldehydes, 4 esters, 15 hydrocarbons, 5 ketones, 2 phenols, and 10 others. The volatile compounds were generally from unsaturated fatty acids, proteins, free amino acids, carbohydrates, triglycerides, or their derivatives, as well as from the photosynthesis and metabolism of vitamins and minerals [27].

Acids in this study were similar to those described by other researchers, for example References [40,41], who demonstrated that cis-vaccenic acid and oleic acid can be found in rice. Acids were probably produced by hydrolysis and oxidation of the rice lipids [6]. Alcohols were probably generated mainly by the thermal oxidation of lipids and by the degradation of carbohydrates [42]. However, alcohols had a smaller influence on odor profile compared to aldehydes and ketones due to their higher odor thresholds [10].

Aldehydes are the most important factor affecting the aroma profile of rice [25] and were mainly formed by the oxidation of amino acids and unsaturated fatty acids [43]. Among 9 aldehydes, hexanal (a lipid oxidation marker in rice) and nonanal have been identified as major aroma-active compounds of jasmine rice [27]. These two compounds are derived from oleic acid and linoleic acid via lipid oxidation caused by grinding [25,44]. It has been reported that hexanal generates mainly fruit and herbaceous aromas at low concentrations, but it causes an unpleasant odor through oil oxidation at high concentrations [45]. A similar situation has been seen in the study of nonanal, where it presented a pleasant odor of citrus and rose at low concentrations but an unpleasant odor at high concentrations [46,47]. Verma and Srivastav [48] regarded hexanal as a key volatile compound responsible for off-flavor. Compared to CR counterparts, AWR had a lower hexanal content (0.5 ± 0.0) but not a low nonanal content (8.0 ± 0.3), suggesting that AWR had a milder aroma profile to those of the CRs.

In rice, hydrocarbons are synthetized in the cuticle and epicuticle [49]. Among the 15 hydrocarbons identified here, straight-chain and cyclic alkanes are thought to be derived from the decarboxylation of long-chain fatty acids [50]. The alkanes have nearly no effects on the aroma profile due to their high thresholds [51]. Among all volatile compounds, 8,8,9-trimethyl-deca-3,5-diene-2,7-dione, one of the ketones, was the most dominant volatile constituent in AWR. The oxidative degradation of unsaturated fatty acids can also contribute to the formation of ketones. Dihydro-5-pentyl-2(3h)-furanone might contribute to the ‘fruity, floral’ odor, while cyclopentadecanone can provide a musk fragrance [31,52]. It has also been noted [53] that 2-tridecanone can be detected in AWR, and this has an odor description of oily and nutty. Phenols only accounted for a small proportion of the volatile compounds. Only two phenols were found here in rices, which is consistent with an earlier report [54] that mentioned that valuable phenolic compounds were mainly found in rice bran. However, phenols with quite low odor thresholds have been reported to make some contribution to rice aroma [25]. Fukuda et al. [55] also found dodecamethyl-cyclohexasiloxane, and cyclooctasiloxane, hexadecamethyl among 10 other compounds, in cooked rice.

### 3.8. Correlations between Chemical Compositions, Molecular Structural Parameters, and Broken Grains

Supplementary Table S2 gives the correlation coefficients between chemical compositions, molecular structural parameters, and broken grains. No significant correlations were obtained between chemical compositions and broken grains because the ranges of starch, protein, and ACs were not very wide. AWR exhibited a higher percentage of broken grains after cooking, which might be affected by the starch molecular structural parameters. Interestingly, *β*_Ap,v_ had significantly negative correlations with broken grain (%).

### 3.9. Correlations between Molecular Structural Parameters, Volatile Compounds, and Sensory Properties

Table 5 gives the correlation coefficients between starch structure and rice sensory measured by panelists. AWR is special case because of lower *β*_Ap,v_, *β*_Am,2_, and *β*_Am,3_, but higher *h*_Ap,v_ and *β*_Am,1_, values than those of commercial ones. These structural differences might contribute indirectly to the variations of rice sensory attributes in aroma (Figure 1A,B), if one hypothesizes that different starch molecular structures might limit the release of aroma. This hypothesis is obtained by noting that, as shown in Table 5, there are some significant relationships between starch molecular fine structure parameters and aroma attributes. For example, the lower *β*_Am,2_ and *β*_Am,3_ in AWR means that the genes controlling the medium and long Am chains in AWR result in chains that are longer compared to those of CRs; these chains will form larger and looser cells in the gel network and more space for the release of volatile compounds, resulting in higher aroma intensity.

The AWR had a *disintegration* texture similar to that of Doongara rice. The *disintegration* texture is negatively correlated with *h*_Ap,i_ (*p* < 0.01). This negative correlation (r = −0.755) indicated that a rice variety containing more Ap short chains tended to have a lower *disintegration* texture. This might be explained by the fact that more Ap short chains can form more stable double helices [56], making it difficult to break down the sample in the mouth when chewing, thereby decreasing the disintegration value. In addition, the AWR had a *cohesiveness* and *stickiness* texture, similar to that of Paella rice. The *h*_Ap,*i*_ and *h*_Ap,*iii*_ had significant positive correlations with both cohesiveness and stickiness.

Supplementary Table S3 summarizes the correlation coefficients between rice sensory measured by panelists and relative contents of volatile compounds. In general, AWR had *raw cookie dough*, *brown bread*, *sulfurous*, *eggy*, *earthy*, and *root vegetable* aroma, as seen in Figure 1A,B. AWR had less *resinous* and *plastic* aroma. The *resinous* aroma was significantly positively correlated with 2-heptenal but negatively correlated with nonadecane, 2h-pyran, tetrahydro-2-(12-pentadecynyloxy)-, and estra-1,3,5(10)-trien-17β-ol. The *plastic* aroma had significantly positive correlations with 2-myristynoyl pantetheine but negative correlations with cis-7-hexadecenoic acid, and estra-1,3,5(10)-trien-17β-ol. 

## 4. Conclusions

An Australian wild rice was compared with common domesticated rices with regard to starch molecular fine structure and the volatile compounds of cooked rices, these together controlling the sensory properties of polished cooked rice. This is potentially useful because the AWR might have both acceptable sensory properties and nutritional advantages due to its slow digestion. To be specific, AWR had different color parameters (lower L*, and *h*°, but higher a*, b*, and C*) for both in raw and cooked polished rices compared to those of CRs. The aroma of the wild rice was complex, such as that of *raw cookie dough*, *cereal* with *brown bread* notes, and some *sulfurous*, *eggy*, *earthy*, and *root vegetable* notes. AWR had less *resinous* aroma and *plastic* aroma compared to those of CRs. The *resinous* aroma was affected by 2-heptenal, nonadecane, 2h-pyran, tetrahydro-2-(12-pentadecynyloxy)-, and estra-1,3,5(10)-trien-17β-ol, and *plastic* aroma was influenced by 2-myristynoyl pantetheine, cis-7-hexadecenoic acid, and estra-1,3,5(10)-trien-17β-ol. For texture, AWR was *cohesive* and *sticky*, neither *fluffy* nor very *chewy*, and *soft* and acceptable *disintegration* in the mouth. AWR had a *disintegrating* texture similar to that of Doongara rice, and this property was caused by the amounts of Ap short chains. Additionally, the flavor of the AWR was complex, such as *cereal/porridge*, with strong *earthy* and *brown bread* flavors. AWR had less *bitter*, *cardboard*, *chemical*, and *plastic* flavor compared to those of CRs. In short, AWR had significantly different but acceptable sensory characteristics compared to CR varieties. This study indicates that AWR has the potential for commercialization as a healthier but palatable rice grain.

## Figures and Tables

**Table 1:** Details of the source, chemical compositions, and minimum cooking times of varieties.

Rice Varieties	Species	Product Details	Country of Origin	Total Starch Content (%) ^1^	Total Crude Protein Content (%) ^1^	Amylose Content (%) ^2^	Minimum Cooking Time (min)
Australian Wild Rice	Wild rice	Harvested by hand from north of Cairns, Queensland (May 2019)	Australia	78.0 ± 0.2 ^a,b^	11.9 ± 0.2 ^b^	24.8 ± 0.4 ^a^	17
Sushi	Japonica	SUNRICE Sushi rice, Japanese style	Australia	79.1 ± 1.6 ^a^	10.0 ± 0.0 ^f^	16.4 ± 0.0 ^d^	15
Doongara	Japonica	SUNRICE Doongara Clever Rice (Low-GI white)	Australia	79.4 ± 1.1 ^a^	11.6 ± 0.2 ^b,c^	22.9 ± 0.2 ^b^	20
Sona Masoori	Indica	KATOOMBA Premium Andhra Sona Masoori rice	India	78.4 ± 0.2 ^a,b^	10.5 ± 0.1 ^e^	24.8 ± 0.5 ^a^	12
Ponni Raw	Indica	PATTU Premium Ponni raw rice	India	75.9 ± 2.5 ^b^	13.2 ± 0.2 ^a^	25.5 ± 0.1 ^a^	12
Paella	Japonica	ARROZ La Marjal, Especial Paellas rice	Spain	77.8 ± 0.6 ^a,b^	11.5 ± 0.2 ^c^	16.1 ± 0.2 ^d^	17
Long grain	Indica	SUNRICE Long Grain, White rice	Thailand	79.0 ± 0.8 ^a^	11.1 ± 0.1 ^d^	25.4 ± 0.0 ^a^	18
Australian medium grain	Japonica	SUNRICE Australian Medium Grain, Calrose rice	Australia	79.0 ± 1.0 ^a^	10.6 ± 0.2 ^e^	17.4 ± 0.5 ^c^	16

^1^ Total starch content and total crude protein content are expressed on dry basis; ^2^ amylose content is expressed on a basis of total starch; values are the means of replicate (*n* = 2) ± SD. Means followed by the same letters did not differ significantly (*p* < 0.05); commercial rices were Sushi, Doongara, Sona Masoori, Ponni Raw, Paella, Long grain, and Australian medium grain.

**Table 2:** Details of physical properties of rice grains ^1^.

Rice Varieties	Length (mm)		Width (mm)		Length/Width		Color—Raw					Color—Cooked					BG ^2^
	Raw	Cooked	Raw	Cooked	Raw	Cooked	L*	a*	b*	C*	*h*°	L*	a*	b*	C*	*h*°	
Australian wild rice	5.9 ± 0.5 ^c^	7.6 ± 0.8 ^e^	1.8 ± 0.2 ^e^	2.6 ± 0.2 ^c^	3.4 ± 0.3 ^b,c^	3.0 ± 0.4 ^c^	63.8 ± 0.9 ^e^	5.8 ± 0.2 ^a^	15.8 ± 0.5 ^a^	16.5 ± 0.3 ^a^	69.5 ± 0.5 ^e^	68.6 ± 1.8 ^d^	4.4 ± 0.1 ^a^	9.2 ± 0.5 ^a^	10.2 ± 0.5 ^a^	64.4 ± 0.5 ^e^	45
Sushi	4.7 ± 0.2 ^f^	7.8 ± 0.5 ^d,e^	2.4 ± 0.1 ^b^	3.3 ± 0.4 ^a^	2.0 ± 0.1 ^f^	2.4 ± 0.4 ^e^	68.6 ± 2.2 ^d^	−1.1 ± 0.1 ^c^	12.7 ± 0.6 ^c,d^	12.7 ± 0.6 ^c,d^	95.1 ± 0.4 ^b^	74.2 ± 1.4 ^c^	−1.4 ± 0.1 ^c^	7.7 ± 0.3 ^b^	7.8 ± 0.3 ^b^	100.4 ± 0.8 ^c^	2
Doongara	6.5 ± 0.5 ^b^	8.9 ± 0.7 ^b^	1.9 ± 0.1 ^d^	2.7 ± 0.2 ^c^	3.4 ± 0.2 ^b^	3.3 ± 0.3 ^c^	75.9 ± 1.3 ^a,b^	−1.1 ± 0.0 ^c^	10.7 ± 0.5 ^e^	10.7 ± 0.5 ^e^	96.1 ± 0.2 ^a^	80.1 ± 5.0 ^b^	−1.3 ± 0.1 ^c^	6.7 ± 0.7 ^c^	7.1 ± 0.1 ^c^	101.3 ± 2.0 ^b,c^	10
Sona Masoori	5.3 ± 0.2 ^e^	8.7 ± 0.7 ^b,c^	1.6 ± 0.1 ^f^	2.2 ± 0.2 ^d^	3.3 ± 0.2 ^c^	4.1 ± 0.5 ^a^	75.6 ± 1.8 ^a,b^	−0.7 ± 0.1 ^b^	13.5 ± 0.5 ^b,c^	13.5 ± 0.5 ^b,c^	93.0 ± 0.2 ^d^	72.0 ± 1.2 ^c,d^	−1.5 ± 0.1 ^c^	6.6 ± 0.5 ^c^	6.8 ± 0.5 ^c,d^	103.2 ± 1.6 ^a,b^	1
Ponni Raw	5.3 ± 0.2 ^e^	8.3 ± 0.7 ^b,c,d^	1.8 ± 0.1 ^e^	2.3 ± 0.3 ^d^	2.9 ± 0.2 ^d^	3.6 ± 0.6 ^b^	73.5 ± 1.1 ^b^	−0.9 ± 0.1 ^b^	11.9 ± 0.3 ^d^	12.0 ± 0.3 ^d^	94.1 ± 0.5 ^c^	84.9 ± 1.1 ^a^	−0.8 ± 0.2 ^b^	8.3 ± 0.6 ^b^	8.4 ± 0.6 ^b^	95.8 ± 1.8 ^d^	6
Paella	5.7 ± 0.2 ^c,d^	10.0 ± 1.3 ^a^	2.8 ± 0.1 ^a^	3.3 ± 0.4 ^a^	2.0 ± 0.1 ^f^	3.1 ± 0.5 ^c^	77.7 ± 0.8 ^a^	−0.7 ± 0.1 ^b^	10.7 ± 0.5 ^e^	10.7 ± 0.5 ^e^	94.0 ± 0.3 ^c^	73.1 ± 1.1 ^c^	−1.4 ± 0.0 ^c^	6.1 ± 0.4 ^c^	6.3 ± 0.4 ^d^	102.5 ± 0.9 ^a,b,c^	7
Long grain	7.3 ± 0.4 ^a^	10.4 ± 1.4 ^a^	2.0 ± 0.1 ^c^	2.7 ± 0.2 ^c^	3.6 ± 0.2 ^a^	3.9 ± 0.6 ^a,b^	71.2 ± 1.1 ^c^	−0.9 ± 0.1 ^b^	13.3 ± 1.0 ^b,c^	13.3 ± 1.0 ^b,c^	93.7 ± 0.2 ^c^	71.7 ± 1.2 ^c,d^	−1.4 ± 0.0 ^c^	5.2 ± 0.1 ^d^	5.3 ± 0.1 ^e^	105.1 ± 0.7 ^a^	28
Australian medium grain	5.6 ± 0.3 ^d^	8.2 ± 0.6 ^c,d,e^	2.4 ± 0.1 ^b^	3.1 ± 0.3 ^b^	2.3 ± 0.2 ^e^	2.7 ± 0.4 ^d^	70.9 ± 0.7 ^c,d^	−1.4 ± 0.1 ^d^	14.1 ± 0.3 ^b^	14.2 ± 0.3 ^b^	95.6 ± 0.3 ^a,b^	74.7 ± 0.5 ^c^	−1.5 ± 0.2 ^c^	6.7 ± 0.2 ^c^	6.9 ± 0.2 ^c,d^	102.8 ± 1.8 ^a,b,c^	31

^1^ Values for dimensions and color parameters (L*, a*, b*, C*, *h*°) and percentage of broken grain for cooked polished rice samples; values are the means of replicates (*n* = 20 for dimension, and *n* = 3 for color) ± SD. Means followed by the same letters did not differ significantly (*p* < 0.05). ^2^ BG (%), Broken grain (%).

**Table 3:** Summary of sensory attribute terms, their corresponding definitions, and statistical analysis ^1^.

Attribute	Definition	Sample (*n* = 8)	Panelist (*n* = 11)	Replicate (*n* = 3)	Sample × Panellist	Sample × Replicate	Panellist × Replicate
***Aroma***							
Aroma intensity	Overall aroma intensity of sample.	5 **	5 **	1 ^ns^	2 **	2 **	1 ^ns^
***Texture***							
Fluffiness	Light fluffy nature of sample when moved with a spoon, grains separated and light.	42 **	5 **	2 ^ns^	3 **	2 **	1 ^ns^
Cohesiveness	How sample sticks to itself as a cohesive mass.	49 **	7 **	1 ^ns^	2 **	3 **	1 ^ns^
Firmness	Hardness or firmness of sample on first chew.	14 **	2 ^ns^	2 ^ns^	2 **	1 ^ns^	1 ^ns^
Stickiness	Glutinous sensation perceived where sample readily sticks both to itself and to oral surfaces.	35 **	1 ^ns^	0 ^ns^	2 **	4 **	1 ^ns^
Chewiness	Amount of chewing required to break down sample.	29 **	2 ^ns^	11 *	1 ^ns^	1 ^ns^	1 ^ns^
Disintegration	How readily sample breaks down in mouth when chewing, disappearing and disintegrating quickly.	9 **	4 **	11 *	2 **	1 ^ns^	1 ^ns^
***Flavor***							
Flavor intensity	Overall flavor intensity.	3 **	3 **	1 ^ns^	3 **	2 **	1 ^ns^

^1^ Statistically significant F ratios indicated by ** (*p* < 0.01), * (*p* < 0.05), ^ns^ not significant (*p* > 0.05). A hybrid of scaled attributes (attributes which were listed here) and “Check-All-That-Apply” attributes (attributes which were not listed here but mentioned in Section 2.7, in the sensory evaluation part) were used in this study.

**Table 4:** Average molecular sizes (nm) of whole ($\overset{—}{R}$_h_) starch molecules, amylose ($\overset{—}{R}$_h,AM_), and amylopectin ($\overset{—}{R}$_h,Ap_) extracted from Australian wild rice and commercial rices ^1^.

Rice Varieties	$\overset{—}{R}$ _h_	$\overset{—}{R}$ _h,AM_	$\overset{—}{R}$ _h,Ap_
Australian Wild Rice	34.9 ± 0.1 ^c,d^	12.8 ± 0.0 ^e^	86.2 ± 0.2 ^c^
Sushi	38.6 ± 0.0 ^b,c^	14.1 ± 0.1 ^b^	98.7 ± 1.5 ^b^
Doongara	36.2 ± 2.8 ^b,c,d^	14.1 ± 0.0 ^b^	96.2 ± 3.2 ^b^
Sona Masoori	38.6 ± 3.5 ^b,c^	14.1 ± 0.0 ^b^	100.9 ± 7.3 ^b^
Ponni Raw	39.5 ± 2.4 ^b^	14.2 ± 0.2 ^b^	117.6 ± 2.3 ^a^
Paella	44.9 ± 1.3 ^a^	13.4 ± 0.1 ^c^	112.8 ± 2.5 ^a^
Long grain	32.9 ± 0.0 ^d^	13.0 ± 0.0 ^d^	84.8 ± 0.6 ^c^
Australian Medium grain	36.2 ± 2.9 ^b,c,d^	14.6 ± 0.0 ^a^	100.4 ± 2.9 ^b^

^1^ Values are the means of two replicates ± SD. Means followed by the same letters do not differ significantly (*p* < 0.05); commercial rices contain Sushi, Doongara, Sona Masoori, Ponni Raw, Paella, Long grain, and Australian Medium grain.

**Table 5:** Pearson correlation between rice sensory measured by panelists and starch structural parameters (*n* = 8) ^1^.

		Ap CLD Fitting Parameters									Am CLD Fitting Parameters							Branched SEC		
		*β*_Ap,i_	*β*_Ap,ii_	*β*_Ap,iii_	*β*_Ap,iv_	*β*_Ap,v_	*β*_Ap,vi_	*h*_Ap,i_	*h*_Ap,iii_	*h*_Ap,v_	Am content	*β*_Am,1_	*β*_Am,2_	*β*_Am,3_	*h*_Am,1_	*h*_Am,2_	*h*_Am,3_	*R*_h_	*R*_h,Am_	*R*_h,Ap_
***Aroma***	Aroma intensity	0.082	−0.267	−0.226	−0.541	−0.587	0.588	−0.196	0.090	0.458	0.478	−0.01	−0.769 *	−0.750 *	0.147	0.451	0.454	−0.200	−0.641	−0.304
	Sulfurous	0.052	−0.084	0.299	−0.427	−0.479	0.720 *	0.152	0.340	0.262	0.104	0.281	−0.756 *	−0.687	−0.662	−0.133	0.452	0.146	−0.166	0.120
	Eggy	0.266	−0.179	0.454	−0.242	−0.007	0.132	0.165	0.529	0.199	0.084	−0.093	−0.383	−0.297	−0.393	0.163	−0.056	−0.234	0.143	−0.062
	Green vegetable	−0.572	0.775 *	−0.066	0.286	0.027	0.616	0.525	0.523	0.097	−0.638	0.697	−0.089	−0.072	−0.591	−0.725 *	−0.195	0.724 *	−0.289	0.270
	Root vegetables	−0.669	0.753 *	−0.045	0.132	−0.096	0.632	0.629	0.354	−0.012	−0.610	0.578	−0.103	−0.115	−0.415	−0.715 *	−0.141	0.735 *	−0.236	0.448
	Sweet caramelized	−0.412	0.033	−0.578	0.084	−0.381	−0.075	0.228	0.269	0.500	0.150	0.190	0.041	−0.082	0.527	0.526	−0.197	−0.777 *	−0.415	−0.736 *
	Brown bread	−0.126	−0.147	0.299	−0.370	−0.075	−0.124	0.235	0.020	−0.244	−0.218	0.200	0.219	0.214	−0.509	−0.145	−0.119	−0.336	0.316	−0.337
	Earthy	−0.544	0.555	−0.226	0.109	−0.386	0.812 *	0.341	0.261	0.215	−0.348	0.762 *	−0.347	−0.363	−0.535	−0.603	0.251	0.622	−0.451	0.209
	Cereal/Porridge	0.219	−0.050	0.373	0.342	0.663	−0.817 *	0.005	−0.219	−0.526	−0.239	−0.368	0.749 *	0.754 *	0.060	−0.147	−0.446	−0.043	0.779 *	0.252
	Fragrant rice	0.737 *	−0.811 *	0.011	−0.355	0.078	−0.547	−0.749 *	−0.560	−0.069	0.746 *	−0.856 **	−0.036	−0.013	0.734 *	0.835 **	0.218	−0.581	0.096	−0.261
	Resinous	0.304	−0.383	0.547	−0.808 *	0.232	−0.188	−0.139	−0.360	−0.601	−0.023	−0.485	0.097	0.183	−0.150	0.018	−0.098	0.003	0.333	0.043
	Plastic	0.379	−0.216	0.088	−0.210	0.168	−0.023	−0.531	−0.678	−0.404	0.204	−0.433	−0.037	0.015	0.225	−0.076	0.334	0.519	0.045	0.484
	Raw cookie dough	−0.220	−0.020	−0.353	0.006	−0.718 *	0.566	0.108	0.413	0.701	0.246	0.584	−0.579	−0.626	−0.261	0.186	0.421	−0.368	−0.508	−0.482
	Cardboard	0.240	−0.155	0.686	−0.163	0.320	−0.326	0.035	−0.478	−0.744 *	−0.210	−0.311	0.334	0.378	−0.357	−0.470	0.044	0.376	0.832 *	0.711 *
	Chemical	0.212	−0.274	−0.326	−0.182	0.012	−0.189	−0.466	−0.441	−0.014	0.392	−0.462	0.002	−0.016	0.747 *	0.483	0.088	−0.152	−0.284	−0.159
***Texture***	Fluffiness	0.582	−0.632	−0.124	−0.175	−0.150	−0.246	−0.760 *	−0.831 *	−0.106	0.726 *	−0.648	−0.165	−0.176	0.682	0.488	0.605	−0.100	0.031	0.212
	Cohesiveness	−0.564	0.614	0.157	0.120	0.109	0.312	0.754 *	0.825 *	0.106	−0.708 *	0.652	0.094	0.113	−0.730 *	−0.506	−0.546	0.139	−0.041	−0.172
	Firmness	−0.739 *	0.693	−0.138	0.476	0.234	−0.182	0.716 *	0.241	−0.186	−0.703	0.378	0.638	0.543	0.012	−0.490	−0.636	0.166	0.175	0.137
	Stickiness	−0.622	0.653	0.077	0.180	0.114	0.265	0.804 *	0.886 **	0.171	−0.706	0.640	0.131	0.133	−0.603	−0.427	−0.632	0.050	−0.086	−0.238
	Chewiness	−0.821 *	0.834 *	−0.023	0.474	0.223	0.012	0.903 **	0.513	−0.113	−0.850 **	0.552	0.517	0.448	−0.278	−0.644	−0.690	0.273	0.155	0.176
	Disintegration	0.654	−0.623	−0.063	−0.382	−0.233	0.167	−0.755 *	−0.232	0.268	0.699	−0.313	−0.576	−0.501	0.124	0.549	0.579	−0.209	−0.335	−0.291
***Flavor***	Flavor intensity	−0.338	0.218	−0.392	−0.193	−0.651	0.875 **	0.084	0.140	0.446	0.126	0.432	−0.696	−0.718 *	−0.058	−0.065	0.479	0.301	−0.731 *	0.029
	Root vegetables	−0.663	0.645	−0.140	−0.030	−0.249	0.731 *	0.535	0.362	0.095	−0.513	0.668	−0.216	−0.227	−0.469	−0.598	−0.044	0.578	−0.409	0.171
	Sweet caramelized	−0.079	0.227	0.313	0.283	0.545	−0.439	0.387	0.535	−0.121	−0.480	0.095	0.513	0.543	−0.352	−0.157	−0.725 *	−0.220	0.415	−0.236
	Brown bread	−0.267	0.172	0.048	0.107	−0.186	0.198	0.383	0.546	0.278	−0.237	0.552	−0.065	−0.075	−0.588	−0.167	−0.103	−0.240	−0.012	−0.355
	Earthy	−0.602	0.527	−0.363	0.024	−0.566	0.937 **	0.407	0.485	0.493	−0.217	0.780 *	−0.556	−0.583	−0.393	−0.343	0.234	0.370	−0.690	−0.033
	Cereal/Porridge	−0.128	−0.002	−0.170	0.221	0.022	−0.273	0.157	0.436	0.311	−0.058	0.245	0.230	0.191	−0.053	0.265	−0.348	−0.661	−0.030	−0.717 *
	Fragrant rice	0.225	−0.380	−0.256	−0.083	0.100	−0.505	−0.279	0.057	0.229	0.350	−0.344	0.180	0.157	0.573	0.767*	−0.286	−0.849 **	−0.144	−0.801 *
	Resinous	−0.297	−0.009	−0.377	−0.369	−0.908 **	0.888 **	0.058	0.090	0.531	0.304	0.459	−0.814 *	−0.856 **	−0.109	0.093	0.665	−0.001	−0.703	−0.155
	Plastic	0.428	−0.252	0.304	−0.101	0.224	−0.213	−0.362	−0.669	−0.488	0.205	−0.602	0.043	0.081	0.281	−0.084	0.281	0.452	0.393	0.767 *
	Cardboard	0.263	−0.470	0.250	−0.521	−0.507	0.255	−0.184	−0.477	−0.104	0.448	−0.263	−0.489	−0.487	−0.005	0.103	0.697	0.019	0.146	0.406
	Chemical	0.340	−0.327	−0.096	−0.188	−0.200	0.085	−0.454	−0.559	−0.033	0.517	−0.485	−0.339	−0.339	0.552	0.280	0.515	0.183	−0.119	0.419
	Bitter	0.380	−0.346	0.118	−0.522	−0.147	0.264	−0.473	−0.532	−0.202	0.369	−0.391	−0.413	−0.350	0.139	0.088	0.514	0.355	−0.136	0.352

^1,^* Correlation is significant at the 0.05 level (two-tailed); ** Correlation is significant at the 0.01 level (two-tailed).

## Data Availability

The datasets generated for this study are available on request to the corresponding author.

## Supplementary Materials

The following are available online at https://www.mdpi.com/article/10.3390/foods11040511/s1, Figure S1: Typical starch digestion curves, model-fit (Parallel-fitting and Sequential-fitting) curves, and LOS plots for cooked AWR compared to CRs (Sushi, Doongara, Sona Masoori, Ponni Raw, Paella, Long grain, and Australian Medium grain). Rep1 and rep2 are two replicates; Figure S2: Comparison of in vitro digestibility parameters of AWR and CRs. (A)–(B) The parameters from Parallel-fitting model; (C)–(D) The parameters from Sequential-fitting model. Blue and orange: AWR and CRs (Sushi, Doongara, Sona Masoori, Ponni Raw, Paella, Long grain, and Australian Medium grain), respectively. All data were from duplicate measurements. The same letters mean not significant difference (*p* < 0.05). *k* is the digestion rate coefficient of starch, and *C*_∞_ is the percentage of starch digested by the end of reaction time; Figure S3: (A)–(H) The visual pictures of AWR and CRs (Sushi, Doongara, Sona Masoori, Ponni Raw, Paella, Long grain, and Australian Medium grain), respectively; Figure S4: Fitting results of Ap (a) and Am (b) CLD for AWR and CRs (Sushi, Doongara, Sona Masoori, Ponni Raw, Paella, Long grain, and Australian Medium grain). Table S1: Volatile component identified and semi-quantified by HS-SPME-GC/MS among AWR and CR grains; Table S2: Pearson correlations between chemical compositions, molecular structural parameters, and broken grains (*n* = 8); Table S3: Pearson correlations between rice sensory measured by panelists and relative contents of volatile compounds (*n* = 8).

## Abbreviations

Am, amylose; Ap, amylopectin; AWR, Australian wild rice; CRs, commercial rices; CLD, chain length distribution; DP, degree of polymerization; SEC, size-exclusion chromatography; FACE, fluorophore-assisted capillary electrophoresis; HS-SPME, headspace solid-phase microextraction; GC-MS, gas chromatography-mass spectrometry.

## References

[1] Huang L., Sreenivasulu N., Liu Q. (2020). Waxy editing: Old meets new. Trends Plant Sci..

[2] Brozynska M., Copetti D., Furtado A., Wing R.A., Crayn D., Fox G., Ishikawa R., Henry R.J. (2017). Sequencing of Australian wild rice genomes reveals ancestral relationships with domesticated rice. Plant Biotechnol. J..

[3] Zhao Y., Henry R.J., Gilbert R.G. (2021). Starch structure-property relations in Australian wild rices compared to domesticated rices. Carbohydr. Polym..

[4] Tikapunya T., Henry R.J., Smyth H. (2018). Evaluating the sensory properties of unpolished Australian wild rice. Food Res. Int..

[5] Tao K., Yu W., Gilbert R.G. (2019). High-amylose rice: Starch molecular structural features controlling cooked rice texture and preference. Carbohydr. Polym..

[6] Yuan B., Zhao C., Yan M., Huang D., David Julian M., Huang Z., Cao C. (2019). Influence of gene regulation on rice quality: Impact of storage temperature and humidity on flavor profile. Food Chem..

[7] Belitz H.-D., Grosch W., Schieberle P., Belitz H.-D., Grosch W., Schieberle P. (2004). Aroma compounds. Food Chemistry.

[8] Li H., Prakash S., Nicholson T.M., Fitzgerald M.A., Gilbert R.G. (2016). Instrumental measurement of cooked rice texture by dynamic rheological testing and its relation to the fine structure of rice starch. Carbohydr. Polym..

[9] Fan N., Shewan H.M., Smyth H.E., Yakubov G.E., Stokes J.R. (2021). Dynamic tribology protocol (DTP): Response of salivary pellicle to dairy protein interactions validated against sensory perception. Food Hydrocoll..

[10] Gao C., Li Y., Pan Q., Fan M., Wang L., Qian H. (2021). Analysis of the key aroma volatile compounds in rice bran during storage and processing via HS-SPME GC/MS. J. Cereal Sci..

[11] Wu A.C., Morell M.K., Gilbert R.G. (2013). A parameterized model of amylopectin synthesis provides key insights into the synthesis of granular starch. PLoS ONE.

[12] Nada S.S., Zou W., Li C., Gilbert R.G. (2017). Parameterizing amylose chain-length distributions for biosynthesis-structure-property relations. Anal. Bioanal. Chem..

[13] Zhao Y., Tan X., Wu G., Gilbert R.G. (2020). Using molecular fine structure to identify optimal methods of extracting starch. Starch-Stärke.

[14] Mohapatra D., Bal S. (2006). Cooking quality and instrumental textural attributes of cooked rice for different milling fractions. J. Food Eng..

[15] Meullenet J.-F., Champagne E.T., Bett K.L., McClung A.M., Kauffmann D. (2000). Instrumental assessment of cooked rice texture characteristics: A method for breeders. Cereal Chem..

[16] Vilaplana F., Gilbert R.G. (2010). Characterization of branched polysaccharides using multiple-detection size separation techniques. J. Sep. Sci..

[17] Cave R.A., Seabrook S.A., Gidley M.J., Gilbert R.G. (2009). Characterization of starch by size-exclusion chromatography: The limitations imposed by shear scission. Biomacromolecules.

[18] Wu A.C., Li E., Gilbert R.G. (2014). Exploring extraction/dissolution procedures for analysis of starch chain-length distributions. Carbohydr. Polym..

[19] Setyaningsih W., Majchrzak T., Dymerski T., Namieśnik J., Palma M. (2019). Key-marker volatile compounds in aromatic rice (*Oryza Sativa*) Grains: An HS-SPME extraction method combined with GC× GC-TOFMS. Molecules.

[20] Li H., Li X., Zhang C.h., Wang J.z., Tang C.h., Chen L.l. (2016). Flavor compounds and sensory profiles of a novel Chinese marinated chicken. J. Sci. Food Agric..

[21] Li H., Dhital S., Gidley M.J., Gilbert R.G. (2019). A more general approach to fitting digestion kinetics of starch in food. Carbohydr. Polym..

[22] Yu W., Tao K., Gilbert R.G. (2018). Improved methodology for analyzing relations between starch digestion kinetics and molecular structure. Food Chem..

[23] Syahariza Z.A., Li E., Hasjim J. (2010). Extraction and dissolution of starch from rice and sorghum grains for accurate structural analysis. Carbohydr. Polym..

[24] Hasjim J., Li E.P., Dhital S. (2013). Milling of rice grains: Effects of starch/flour structures on gelatinization and pasting properties. Carbohydr. Polym..

[25] Hu X., Lu L., Guo Z., Zhu Z. (2020). Volatile compounds, affecting factors and evaluation methods for rice aroma: A review. Trends Food Sci. Technol..

[26] Van Gemert L. (2003). Compilations of Odour Threshold Values in Air, Water and Other Media.

[27] Zhao Q., Xue Y., Shen Q. (2020). Changes in the major aroma-active compounds and taste components of Jasmine rice during storage. Food Res. Int..

[28] Song G., Zhang M., Zhang Y., Wang H., Chen K., Dai Z., Shen Q. (2019). Development of a 450 nm laser irradiation desorption method for fast headspace solid-phase microextraction of volatiles from krill oil (*Euphausia superba*). Eur. J. Lipid Sci. Technol..

[29] Jia X., Zhou Q., Wang J., Liu C., Huang F., Huang Y. (2019). Identification of key aroma—active compounds in sesame oil from microwaved seeds using E-nose and HS-SPME-GC× GC-TOF/MS. J. Food Biochem..

[30] Migita K., Iiduka T., Tsukamoto K., Sugiura S., Tanaka G., Sakamaki G., Yamamoto Y., Takeshige Y., Miyazawa T., Kojima A. (2017). Retort beef aroma that gives preferable properties to canned beef products and its aroma components. Anim. Sci. J..

[31] Parmar K., Patel J., Sheth N., Selvamuthukumaran M., Pathak Y.V. (2018). Flavor nanotechnology: Recent trends and applications. Flavors for Nutraceutical and Functional Foods.

[32] Feng T., Yang M., Ma B., Zhao Y., Zhuang H., Zhang J., Chen D. (2021). Volatile profiles of two genotype *Agaricus bisporus* species at different growth stages. Food Res. Int..

[33] Wijit N., Prasitwattanaseree S., Mahatheeranont S., Wolschann P., Jiranusornkul S., Nimmanpipug P. (2017). Estimation of retention time in GC/MS of volatile metabolites in fragrant rice using principle components of molecular descriptors. Anal. Sci..

[34] Attar U., Hinge V., Zanan R., Adhav R., Nadaf A. (2017). Identification of aroma volatiles and understanding 2-acetyl-1-pyrroline biosynthetic mechanism in aromatic mung bean (*Vigna radiata* (L.) Wilczek). Physiol. Mol. Biol. Plants.

[35] Beldarrain L.R., Morán L., Sentandreu M.Á., Barron L.J.R., Aldai N. (2022). Effect of ageing time on the volatile compounds from cooked horse meat. Meat Sci..

[36] Dias A.L.B., dos Santos P., Martínez J. (2018). Supercritical CO_2_ technology applied to the production of flavor ester compounds through lipase-catalyzed reaction: A review. J. CO2 Util..

[37] Bu T., Zhou M., Zheng J., Yang P., Song H., Li S., Wu J. (2020). Preparation and characterization of a low-phenylalanine whey hydrolysate using two-step enzymatic hydrolysis and macroporous resin adsorption. LWT—Food Sci. Technol..

[38] Oliveira W.d.S., Monsalve J.O., Nerin C., Padula M., Godoy H.T. (2020). Characterization of odorants from baby bottles by headspace solid phase microextraction coupled to gas chromatography-olfactometry-mass spectrometry. Talanta.

[39] Nishimura O. (1995). Identification of the characteristic odorants in fresh rhizomes of ginger (*Zingiber officinale* Roscoe) using aroma extract dilution analysis and modified multidimensional gas chromatography-mass spectroscopy. J. Agric. Food Chem..

[40] Malathi K., Ramaiah S. (2017). Ethyl iso-allocholate from a medicinal rice Karungkavuni inhibits dihydropteroate synthase in Escherichia coli: A molecular docking and dynamics study. Indian J. Pharm. Sci..

[41] Kitta K., Ebihara M., Iizuka T., Yoshikawa R., Isshiki K., Kawamoto S. (2005). Variations in lipid content and fatty acid composition of major non-glutinous rice cultivars in Japan. J. Food Compos. Anal..

[42] Yan W., Liu Q., Wang Y., Tao T., Liu B., Liu J., Ding C. (2020). Inhibition of lipid and aroma deterioration in rice bran by infrared heating. Food Bioprocess Technol..

[43] Xie J.-C., Sun B.-G., Wang S.-B. (2008). Aromatic constituents from Chinese traditional smoke-cured bacon of Mini-pig. Food Sci. Technol. Int..

[44] Xiao L., Lee J., Zhang G., Ebeler S.E., Wickramasinghe N., Seiber J., Mitchell A.E. (2014). HS-SPME GC/MS characterization of volatiles in raw and dry-roasted almonds (*Prunus dulcis*). Food Chem..

[45] Xu D., Hong Y., Gu Z., Cheng L., Li Z., Li C. (2019). Effect of high pressure steam on the eating quality of cooked rice. LWT—Food Sci. Technol..

[46] Morris W.L., Shepherd T., Verrall S.R., McNicol J.W., Taylor M.A. (2010). Relationships between volatile and non-volatile metabolites and attributes of processed potato flavour. Phytochemistry.

[47] Kim M., Sowndhararajan K., Choi H.J., Park S.J., Kim S. (2019). Olfactory stimulation effect of aldehydes, nonanal, and decanal on the human electroencephalographic activity, according to nostril variation. Biomedicines.

[48] Verma D.K., Srivastav P.P. (2020). A paradigm of volatile aroma compounds in rice and their product with extraction and identification methods: A comprehensive review. Food Res. Int..

[49] Zhang X., Dai Z., Fan X., Liu M., Ma J., Shang W., Liu J., Strappe P., Blanchard C., Zhou Z. (2020). A study on volatile metabolites screening by HS-SPME-GC-MS and HS-GC-IMS for discrimination and characterization of white and yellowed rice. Cereal Chem..

[50] Kunst L., Samuels A.L. (2003). Biosynthesis and secretion of plant cuticular wax. Prog. Lipid Res..

[51] Hui Y.H., Chandan R.C., Clark S., Cross N.A., Dobbs J.C., Hurst W.J., Nollet L.M., Shimoni E., Sinha N.K., Smith E.B. (2007). Handbook of Food Products Manufacturing: Health, Meat, Milk, Poultry, Seafood, and Vegetables.

[52] Maraval I., Mestres C., Pernin K., Ribeyre F., Boulanger R., Guichard E., Gunata Z. (2008). Odor-active compounds in cooked rice cultivars from Camargue (France) analyzed by GC−O and GC−MS. J. Agric. Food Chem..

[53] Dias L., Duarte G., Mariutti L., Bragagnolo N. (2019). Aroma profile of rice varieties by a novel SPME method able to maximize 2-acetyl-1-pyrroline and minimize hexanal extraction. Food Res. Int..

[54] Peanparkdee M., Patrawart J., Iwamoto S. (2020). Physicochemical stability and in vitro bioaccessibility of phenolic compounds and anthocyanins from Thai rice bran extracts. Food Chem..

[55] Fukuda T., Takeda T., Yoshida S. (2014). Comparison of volatiles in cooked rice with various amylose contents. Food Sci. Technol. Res..

[56] Li G., Hemar Y., Zhu F. (2021). Relationships between supramolecular organization and amylopectin fine structure of quinoa starch. Food Hydrocoll..

