# Supplementary material for: Starch Molecular Structural Features and Volatile Compounds Affecting the Sensory Properties of Polished Australian Wild Rice
Source: Foods. 2022 Feb 10;11(4):511. doi: 10.3390/foods11040511 (PMC8871513; doi:10.3390/foods11040511)
Supplement: Supplementary file 1 [file foods-11-00511-s001.zip › foods-1581116-supplementary.pdf]

Table S1. Volatile components identified and semi-quantified by HS-SPME-GC/MS among AWR and CR grains[1, 2].

No	Compound name	Similarity (%)	Category	Retention time (min)	Aroma Description[3-18]	Odour threshold ($ng \cdot L^{-1}$ in air)[3-18]	Australian Wild rice (ng/g)	Sushi (ng/g)	Doongara (ng/g)	Sona Masoori (ng/g)	Ponni Raw (ng/g)	Paella (ng/g)	Long grain (ng/g)	Australian Medium grain (ng/g)
1	cyclotetrasiloxane, octamethyl	87	Other	4.70	-	-	2.4 ± 0.4^{bc}	3.0 ± 0.1^{b}	1.8 ± 0.1^{cd}	3.7 ± 1.0^{a}	2.2 ± 0.1^{c}	2.4 ± 0.0^{bc}	1.5 ± 0.0^{d}	1.3 ± 0.2^{d}
2	undecane	73	Hydrocarbon	4.84	Milky	5600	0.9 ± 0.0^{d}	2.1 ± 0.1^{a}	1.5 ± 0.1^{b}	1.1 ± 0.1^{c}	0.0 ± 0.0^{f}	1.0 ± 0.1^{cd}	0.0 ± 0.0^{f}	0.5 ± 0.0^{e}
3	furfural	78	Aldehyde	4.94	Cooked rice, nutty, roasted, sweet	2800	1.4 ± 0.3^{cd}	1.9 ± 0.1^{a}	1.1 ± 0.2^{de}	1.9 ± 0.0^{a}	1.6 ± 0.0^{bc}	1.7 ± 0.0^{ab}	1.2 ± 0.2^{de}	1.1 ± 0.1^{e}
4	hexanal	79	Aldehyde	5.70	Fruity, grass, green, green tomato, grass-like	1.1	0.0 ± 0.0^{d}	60.7 ± 4.1^{a}	30.7 ± 2.2^{b}	2.1 ± 1.0^{d}	17.2 ± 0.9^{c}	1.6 ± 0.6^{d}	1.6 ± 0.0^{d}	0.8 ± 0.2^{d}
5	3,7,11-trimethyl-1-dodecanol	70	Alcohol	6.07	Rubbery, paint-like	-	0.0 ± 0.0^{c}	27.1 ± 0.8^{a}	27.4 ± 1.6^{a}	1.9 ± 0.9^{c}	16.3 ± 2.3^{b}	1.0 ± 0.0^{c}	0.0 ± 0.0^{c}	0.4 ± 0.0^{c}
6	2-heptenal	61	Aldehyde	6.49	Herbaceous, fatty, green	56	0.0 ± 0.0^{e}	0.9 ± 0.2^{d}	1.6 ± 0.2^{c}	2.4 ± 0.0^{b}	0.0 ± 0.0^{e}	2.8 ± 0.0^{a}	0.8 ± 0.1^{d}	1.6 ± 0.2^{c}
7	dodecanal	68	Aldehyde	6.83	Minty, soapy	33	1.5 ± 0.3^{bc}	1.7 ± 0.2^{abc}	0.0 ± 0.0^{e}	2.0 ± 0.1^{ab}	2.2 ± 0.5^{a}	1.3 ± 0.3^{cd}	1.3 ± 0.3^{cd}	0.9 ± 0.2^{d}
8	dihydro-5-pentyl-2(3h)-furanone	76	Ketone	7.34	Sweet fruity	-	0.0 ± 0.0^{d}	2.6 ± 0.1^{a}	0.0 ± 0.0^{d}	2.6 ± 0.2^{a}	1.2 ± 0.2^{b}	1.4 ± 0.1^{b}	1.4 ± 0.3^{b}	0.6 ± 0.0^{c}
9	dodecane	80	Hydrocarbon	8.16	Gasoline like	770	0.0 ± 0.0^{d}	1.5 ± 0.2^{b}	1.7 ± 0.2^{b}	2.0 ± 0.1^{a}	0.0 ± 0.0^{d}	0.8 ± 0.2^{c}	0.7 ± 0.1^{c}	0.6 ± 0.1^{c}
10	5-tridecene, (z)-	65	Hydrocarbon	8.41	-	-	2.8 ± 0.8^{a}	0.0 ± 0.0^{e}	1.1 ± 0.2^{cd}	1.4 ± 0.1^{c}	0.0 ± 0.0^{e}	2.1 ± 0.2^{b}	0.8 ± 0.1^{d}	0.8 ± 0.2^{d}
11	2-methoxy-phenol	62	Phenol	9.48	Smoky-like	13**	1.5 ± 0.2^{b}	2.5 ± 0.4^{b}	1.9 ± 0.3^{b}	6.1 ± 0.8^{b}	1400.5 ± 19.3^{a}	2.6 ± 0.6^{b}	1.7 ± 0.1^{b}	2.0 ± 0.1^{b}
12	cis-vaccenic acid	72	Acid	10.02	-	-	10.3 ± 0.4^{a}	1.6 ± 0.6^{b}	10.6 ± 0.1^{a}	0.0 ± 0.0^{c}	0.0 ± 0.0^{c}	10.5 ± 0.2^{a}	10.4 ± 0.1^{a}	10.6 ± 0.2^{a}
13	octadecane, 1-(ethenyloxy)-	74	Other	10.38	-	-	5.2 ± 0.1^{b}	7.0 ± 1.0^{a}	4.1 ± 0.4^{c}	5.5 ± 0.1^{b}	4.2 ± 0.3^{c}	2.6 ± 0.2^{d}	2.5 ± 0.3^{d}	2.6 ± 0.2^{d}
14	2,6,10-trimethyl-tetradecane	77	Hydrocarbon	11.25	-	-	6.5 ± 0.7^{a}	0.0 ± 0.0^{e}	0.0 ± 0.0^{e}	5.9 ± 1.1^{a}	1.7 ± 0.2^{d}	4.6 ± 0.1^{b}	3.8 ± 0.2^{b}	2.9 ± 0.2^{c}
15	heptadecane	75	Hydrocarbon	11.70	Floral	-	2.4 ± 0.2^{b}	4.5 ± 0.9^{a}	1.3 ± 0.3^{c}	1.1 ± 0.2^{c}	1.0 ± 0.1^{c}	3.0 ± 0.6^{b}	1.3 ± 0.4^{c}	1.0 ± 0.0^{c}
16	pentadecane	76	Hydrocarbon	12.36	Mild odor	>13000000**	3.7 ± 1.1^{a}	1.5 ± 0.1^{c}	2.9 ± 0.1^{ab}	1.0 ± 0.3^{c}	1.5 ± 0.2^{c}	2.5 ± 0.2^{b}	3.4 ± 0.5^{a}	3.4 ± 0.1^{a}
17	4-methyldecane	67	Hydrocarbon	12.87	Pungent	-	0.0 ± 0.0^{c}	3.5 ± 0.6^{a}	1.7 ± 0.1^{b}	3.2 ± 0.3^{a}	3.4 ± 0.1^{a}	1.5 ± 0.2^{b}	1.4 ± 0.2^{b}	0.0 ± 0.0^{c}
18	cyclopentadecanone	68	Ketone	13.01	Musk fragrance	-	2.8 ± 0.1^{c}	5.6 ± 0.3^{a}	2.5 ± 0.3^{c}	3.7 ± 0.3^{b}	5.6 ± 0.8^{a}	2.3 ± 0.2^{cd}	1.8 ± 0.1^{de}	1.4 ± 0.0^{e}
19	dodecamethyl-cyclohexasiloxane	84	Other	13.43	-	-	2.0 ± 0.0^{b}	1.8 ± 0.1^{bc}	1.3 ± 0.1^{d}	2.0 ± 0.2^{b}	2.5 ± 0.3^{a}	1.4 ± 0.1^{d}	1.6 ± 0.2^{cd}	0.7 ± 0.1^{e}
20	1-octanol	62	Alcohol	14.67	Fatty, metallic,citrus, fruity, floral	22	1.2 ± 0.2^{c}	0.0 ± 0.0^{e}	1.7 ± 0.3^{b}	2.0 ± 0.2^{a}	0.0 ± 0.0^{e}	0.9 ± 0.0^{cd}	1.0 ± 0.0^{c}	0.7 ± 0.1^{d}
21	2-tridecanone	61	Ketone	14.89	Oily, nutty	-	1.3 ± 0.1^{cd}	1.6 ± 0.2^{c}	2.2 ± 0.1^{b}	2.6 ± 0.4^{a}	2.5 ± 0.1^{ab}	1.2 ± 0.0^{d}	1.3 ± 0.1^{cd}	1.1 ± 0.0^{d}
22	nonanal	81	Aldehyde	14.98	Citrus, cucumber, floral, fresh, grass, soapy, fatty, fruity	3.1	2.5 ± 0.3^{a}	2.4 ± 0.2^{a}	2.7 ± 0.0^{a}	1.0 ± 0.2^{b}	0.9 ± 0.1^{b}	2.7 ± 0.0^{a}	2.7 ± 0.1^{a}	2.6 ± 0.2^{a}
23	tetradecane	72	Hydrocarbon	15.32	Gasoline-like, alkane	5000	5.2 ± 0.1^{ab}	7.7 ± 1.4^{a}	4.9 ± 0.9^{ab}	0.0 ± 0.0^{d}	0.0 ± 0.0^{d}	3.5 ± 0.4^{c}	3.2 ± 0.2^{c}	2.7 ± 0.2^{c}
24	heptacosane	78	Hydrocarbon	15.56	-	-	2.3 ± 0.0^{b}	1.9 ± 0.1^{c}	2.6 ± 0.1^{a}	2.8 ± 0.1^{a}	2.7 ± 0.2^{a}	2.4 ± 0.2^{b}	2.2 ± 0.1^{b}	2.2 ± 0.1^{b}
25	benzene, 1,3-bis(1,1-dimethylethyl)	96	Hydrocarbon	15.96	Beany, bad	-	1.1 ± 0.0^{d}	2.7 ± 0.4^{a}	1.7 ± 0.5^{c}	1.9 ± 0.0^{bc}	1.9 ± 0.1^{bc}	1.1 ± 0.1^{d}	1.0 ± 0.1^{d}	2.1 ± 0.0^{b}
26	6-methyl-octadecane	75	Hydrocarbon	16.33	-	10*	1.4 ± 0.0^{a}	0.0 ± 0.0^{e}	1.1 ± 0.2^{c}	1.1 ± 0.1^{c}	0.9 ± 0.0^{d}	1.3 ± 0.0^{ab}	1.2 ± 0.1^{b}	1.2 ± 0.1^{b}
27	1-octen-3-ol	70	Alcohol	17.64	Raw mushroom, straw	2.7	0.0 ± 0.0^{d}	1.4 ± 0.1^{c}	0.0 ± 0.0^{d}	1.8 ± 0.2^{b}	1.6 ± 0.0^{c}	1.8 ± 0.1^{b}	1.5 ± 0.2^{c}	2.3 ± 0.1^{a}
28	2-hexyl-1-octanol	72	Alcohol	17.74	Waxy	-	15.2 ± 0.1^{a}	15.0 ± 0.2^{a}	15.5 ± 2.1^{a}	15.5 ± 0.3^{a}	13.3 ± 1.6^{b}	11.5 ± 0.0^{c}	11.6 ± 0.2^{c}	11.5 ± 0.8^{c}
29	1-eicosanol	63	Alcohol	18.85	-	-	1.3 ± 0.0^{b}	1.6 ± 0.0^{a}	1.2 ± 0.1^{bc}	1.5 ± 0.0^{a}	1.3 ± 0.1^{bc}	1.2 ± 0.2^{bc}	0.9 ± 0.1^{d}	1.1 ± 0.1^{cd}
30	2-decen-1-ol	65	Alcohol	19.02	Waxy, fresh	-	4.4 ± 0.3^{bc}	4.6 ± 0.5^{bc}	4.3 ± 0.2^{c}	5.0 ± 0.5^{ab}	2.2 ± 0.4^{e}	5.3 ± 0.3^{a}	3.7 ± 0.3^{d}	3.1 ± 0.1^{d}
31	2-decenal	69	Aldehyde	19.52	Fatty, waxy, green	2.7	3.9 ± 0.1^{b}	5.9 ± 0.1^{a}	3.5 ± 0.2^{c}	4.1 ± 0.1^{b}	2.2 ± 0.1^{e}	3.1 ± 0.1^{d}	2.4 ± 0.0^{e}	2.9 ± 0.2^{d}

					herbal geranium									
32	2-methyl-1-hexadecanol	73	Alcohol	19.91	Medical, mushroom, rotten	-	1.2 ± 0.0^{c}	1.4 ± 0.2^{c}	1.7 ± 0.0^{b}	1.9 ± 0.1^{a}	1.2 ± 0.2^{c}	0.0 ± 0.0^{e}	0.0 ± 0.0^{e}	0.7 ± 0.2^{d}
33	benzaldehyde	76	Aldehyde	20.34	Nutty, sweet, bitter, almond	85	1.3 ± 0.0^{cd}	0.0 ± 0.0^{e}	1.6 ± 0.1^{cd}	38.9 ± 1.6^{a}	35.1 ± 0.0^{b}	1.4 ± 0.2^{cd}	0.9 ± 0.1^{de}	2.2 ± 0.1^{c}
34	8,8,9-trimethyl-deca-3,5-diene-2,7-dione	61	Ketone	20.45	-	-	32.5 ± 0.9^{b}	37.6 ± 3.0^{a}	38.7 ± 4.5^{a}	0.0 ± 0.0^{d}	0.0 ± 0.0^{d}	31.6 ± 2.7^{b}	25.0 ± 1.2^{c}	29.6 ± 0.2^{b}
35	2-nonenal	71	Aldehyde	21.18	Fatty, woody	0.039	1.6 ± 0.3^{b}	2.4 ± 0.5^{a}	2.5 ± 0.7^{a}	0.9 ± 0.2^{c}	2.0 ± 0.2^{ab}	1.0 ± 0.1^{c}	0.0 ± 0.0^{d}	0.8 ± 0.0^{c}
36	2-myristynoyl pantetheine	63	Other	22.43	-	-	1.8 ± 0.1^{ab}	1.7 ± 0.0^{b}	1.9 ± 0.1^{ab}	2.1 ± 0.0^{a}	2.1 ± 0.2^{a}	2.0 ± 0.2^{a}	1.8 ± 0.1^{ab}	1.7 ± 0.2^{b}
37	nonadecane	79	Hydrocarbon	23.47	Sweet, rosy	-	1.9 ± 0.3^{a}	1.7 ± 0.3^{a}	0.0 ± 0.0^{d}	0.0 ± 0.0^{d}	1.4 ± 0.0^{b}	0.9 ± 0.1^{c}	1.6 ± 0.1^{ab}	1.0 ± 0.1^{c}
38	10-methyl-e-11-tridecen-1-ol propionate	69	Hydrocarbon	23.88	-	-	5.7 ± 0.3^{b}	5.4 ± 0.3^{b}	6.3 ± 0.8^{ab}	7.2 ± 0.9^{a}	5.2 ± 0.0^{b}	2.2 ± 1.3^{c}	2.6 ± 0.5^{c}	0.6 ± 0.1^{d}
39	2-nonen-1-ol	73	Alcohol	24.75	Green type	209	10.8 ± 0.9^{cd}	12.6 ± 0.9^{bc}	9.7 ± 0.2^{d}	13.3 ± 0.7^{ab}	15.0 ± 2.6^{a}	9.3 ± 0.9^{d}	9.1 ± 0.3^{d}	9.4 ± 0.3^{d}
40	oleic acid	73	Acid	25.66	-	-	1.5 ± 0.4^{b}	0.0 ± 0.0^{c}	0.0 ± 0.0^{c}	1.4 ± 0.0^{b}	1.3 ± 0.3^{b}	2.4 ± 0.5^{a}	1.7 ± 0.1^{b}	1.7 ± 0.2^{b}
41	dimethyl silanediol	70	Alcohol	25.94	-	-	3.3 ± 0.7^{b}	2.9 ± 0.6^{bc}	2.5 ± 0.2^{cd}	5.4 ± 0.2^{a}	1.9 ± 0.1^{de}	1.9 ± 0.0^{de}	1.3 ± 0.3^{e}	1.7 ± 0.1^{e}
42	2-butyl-2-octenal	87	Aldehyde	26.22	Sour, fruity, pineapple, lemon	20*	0.0 ± 0.0^{d}	1.8 ± 0.1^{a}	1.6 ± 0.2^{a}	0.0 ± 0.0^{d}	1.1 ± 0.1^{b}	1.6 ± 0.2^{a}	1.6 ± 0.1^{a}	0.8 ± 0.1^{c}
43	cyclooctasiloxane, hexadecamethyl	89	Other	27.37	-	-	1.0 ± 0.1^{c}	1.5 ± 0.4^{b}	1.5 ± 0.2^{b}	1.2 ± 0.1^{bc}	1.9 ± 0.1^{a}	1.4 ± 0.1^{b}	0.0 ± 0.0^{d}	0.0 ± 0.0^{d}
44	geranyl isovalerate	79	Ester	27.52	Rose odor with an apple-pineapple undertone and sweet apple taste	-	2.0 ± 0.1^{cd}	2.8 ± 0.2^{a}	2.5 ± 0.4^{ab}	2.2 ± 0.2^{bc}	2.0 ± 0.1^{cd}	2.9 ± 0.1^{a}	1.5 ± 0.2^{e}	1.6 ± 0.1^{de}
45	naphthalene	60	Other	27.81	Naphthalene	450	0.0 ± 0.0^{d}	1.7 ± 0.1^{b}	0.0 ± 0.0^{d}	1.9 ± 0.2^{a}	1.3 ± 0.1^{c}	0.0 ± 0.0^{d}	1.7 ± 0.1^{ab}	0.0 ± 0.0^{d}
46	z-8-methyl-9-tetradecenoic acid	66	Acid	28.93	-	-	3.0 ± 0.0^{b}	2.3 ± 0.0^{d}	2.7 ± 0.3^{bc}	2.3 ± 0.2^{d}	2.0 ± 0.1^{e}	2.5 ± 0.1^{cd}	2.3 ± 0.1^{d}	3.4 ± 0.1^{a}
47	methoxy-phenyl-oxime	62	Other	30.54	-	-	0.0 ± 0.0^{e}	4.0 ± 0.2^{a}	1.9 ± 0.1^{c}	1.7 ± 0.3^{c}	2.2 ± 0.0^{b}	1.7 ± 0.2^{c}	0.0 ± 0.0^{e}	0.7 ± 0.1^{d}
48	1,2-15,16-diepoxyhexadecane	73	Other	31.20	-	-	3.2 ± 0.5^{bc}	3.9 ± 0.3^{a}	3.1 ± 0.3^{bc}	3.3 ± 0.3^{b}	3.5 ± 0.5^{ab}	2.7 ± 0.1^{cd}	2.5 ± 0.0^{d}	3.5 ± 0.0^{ab}
49	10-heptadecen-8-ynoic acid, methyl ester, (e)-	70	Ester	32.06	-	-	7.7 ± 1.2^{de}	8.6 ± 0.1^{cde}	12.8 ± 1.6^{b}	15.7 ± 2.7^{a}	10.8 ± 0.6^{bc}	9.4 ± 0.2^{cd}	6.6 ± 0.0^{e}	6.9 ± 1.0^{e}
50	cis-7-hexadecenoic acid	70	Acid	32.82	-	-	1.4 ± 0.0^{b}	1.9 ± 0.2^{a}	1.4 ± 0.2^{b}	0.0 ± 0.0^{d}	1.5 ± 0.0^{b}	0.0 ± 0.0^{d}	1.1 ± 0.1^{c}	1.0 ± 0.2^{c}
51	2-hexadecanol	69	Alcohol	33.50	Floral, herbal, green	-	1.5 ± 0.3^{cd}	2.4 ± 0.5^{a}	1.7 ± 0.2^{bcd}	2.0 ± 0.6^{abc}	0.0 ± 0.0^{e}	0.0 ± 0.0^{e}	1.4 ± 0.1^{d}	2.1 ± 0.3^{ab}
52	cholestan-3-ol, 2-methylene-, (3β,5α)-	70	Alcohol	36.57	-	-	1.2 ± 0.1^{e}	1.7 ± 0.1^{b}	1.6 ± 0.2^{bc}	2.0 ± 0.0^{a}	1.3 ± 0.1^{de}	1.4 ± 0.0^{cd}	1.6 ± 0.2^{bc}	1.6 ± 0.1^{bc}
53	2h-pyran, tetrahydro-2-(12-pentadecynyloxy)-	72	Other	37.41	-	-	1.7 ± 0.2^{a}	1.5 ± 0.1^{ab}	0.0 ± 0.0^{f}	1.1 ± 0.2^{cde}	1.3 ± 0.0^{bc}	1.2 ± 0.1^{cd}	1.0 ± 0.0^{e}	1.0 ± 0.1^{de}
54	2-methyl-e,e-3,13-octadecadien-1-ol	70	Alcohol	37.58	-	-	1.7 ± 0.4^{ab}	2.0 ± 0.2^{a}	1.7 ± 0.2^{ab}	1.6 ± 0.2^{b}	1.1 ± 0.1^{c}	0.0 ± 0.0^{d}	0.8 ± 0.0^{c}	0.9 ± 0.1^{c}
55	2,4-di-tert-butylphenol	91	Phenol	44.11	Oxidized, lavander, unpleasant	330000**	1.8 ± 0.2^{a}	1.6 ± 0.1^{ab}	1.3 ± 0.3^{bc}	1.5 ± 0.4^{ab}	1.3 ± 0.1^{bc}	1.0 ± 0.1^{cd}	1.0 ± 0.2^{cd}	0.9 ± 0.1^{d}
56	hexadecanoic acid, methyl ester	73	Ester	50.43	Toasted	-	20.5 ± 3.2^{a}	15.4 ± 1.1^{b}	20.6 ± 1.4^{a}	13.4 ± 2.4^{bc}	12.9 ± 0.7^{bc}	14.0 ± 0.7^{bc}	13.4 ± 0.8^{bc}	11.9 ± 0.3^{c}
57	9,11-octadecadienoic acid, methyl ester, (e,e)-	84	Ester	53.09	-	-	3.1 ± 0.0^{d}	3.7 ± 0.2^{b}	3.3 ± 0.1^{cd}	3.6 ± 0.2^{bc}	3.8 ± 0.2^{b}	5.8 ± 0.1^{a}	3.0 ± 0.4^{d}	2.5 ± 0.1^{e}
58	(e)-2-dodecenal	73	Hydrocarbon	54.53	Fatty, green	-	8.1 ± 0.1^{c}	8.1 ± 0.1^{c}	7.0 ± 0.4^{cde}	10.1 ± 0.9^{b}	11.7 ± 1.5^{a}	6.5 ± 0.0^{de}	7.3 ± 0.4^{cd}	5.9 ± 0.1^{e}
59	tert-hexadecanethiol	74	Alcohol	55.30	Almond shell	-	3.0 ± 0.0^{abc}	3.9 ± 0.2^{a}	3.4 ± 0.3^{abc}	2.9 ± 1.3^{bc}	3.8 ± 0.3^{ab}	3.0 ± 0.2^{abc}	2.8 ± 0.2^{c}	2.7 ± 0.2^{c}
60	hexadecane	75	Hydrocarbon	57.42	Green tea-like, meaty	500	0.8 ± 0.2^{b}	0.0 ± 0.0^{c}	0.7 ± 0.1^{b}	1.0 ± 0.1^{a}	0.8 ± 0.1^{b}	0.0 ± 0.0^{c}	0.7 ± 0.1^{b}	0.6 ± 0.1^{b}
61	6,10-dimethyl-5,9-undecadien-2-one	65	Ketone	61.05	Green, fruit, rose	-	1.9 ± 0.0^{b}	1.7 ± 0.3^{bc}	1.6 ± 0.1^{bc}	1.8 ± 0.3^{bc}	2.2 ± 0.3^{a}	1.7 ± 0.1^{bc}	1.2 ± 0.1^{d}	1.5 ± 0.0^{bc}
62	estra-1,3,5(10)-trien-17β-ol	78	Other	62.76	-	-	1.2 ± 0.0^{a}	1.0 ± 0.1^{b}	0.6 ± 0.1^{d}	0.0 ± 0.0^{e}	1.1 ± 0.0^{ab}	0.0 ± 0.0^{e}	0.8 ± 0.0^{c}	0.6 ± 0.1^{d}

[1] Sample means with different lowercase letters in the same row are significantly different ($p < 0.05$).

[2] The relative contents of volatiles components were semi-quantified by peak areas in the total ion chromatogram using 2-Methyl-3-heptanone as an internal standard

solution.

[3-18] Hu, Lu, Guo, Zhu, (2020); van Gemert, (2011); Zhao, Xue, Shen. (2020); Song, Zhang, Zhang, Wang, Chen, Dai, Shen, (2019); Jia, Zhou, Wang, Liu, Huang, Huang, (2019); Migita, Iiduka, Tsukamoto, Sugiura, Tanaka, Sakamaki, Yamamoto, Takeshige, Miyazawa, Kojima, (2017); Attar, Hinge, Zanan, Adhav, Nadaf, (2017); Feng, Yang, Ma, Zhao, Zhuang, Zhang, Chen, (2021); Parmar, Patel, Sheth, (2018); Wijit, Prasitwattanaseree, Mahatheeranont, Wolschann, Jiranusornkul, Nimmanpipug, (2017); Beldarrain, Morán, Sentandreu, Barron, Aldai, (2022); Dias, dos Santos, Martínez, (2018); Bu, Zhou, Zheng, Yang, Song, Li, Wu, (2020); Oliveira, Monsalve, Nerin, Padula, Godoy, (2020); Nishimura, (1995). Odor threshold values were obtained in air; *: odor threshold value were obtained in water, $ng \cdot g^{-1}$; **: odor threshold values were obtained in mineral oil, $ng \cdot g^{-1}$.

Table S2. Pearson correlations between chemical compositions, molecular structural parameters and broken grains (n=8)[1].

		**Broken grain (%)**
Chemical compositions	Total starch content (%)	0.151
	Total crude protein content (%)	0.107
	Amylose content (%)	0.212
Ap CLD fitting parameters	$\beta_{Ap,i}$	-0.407
	$\beta_{Ap,ii}$	-0.05
	$\beta_{Ap,iii}$	-0.38
	$\beta_{Ap,iv}$	-0.259
	$\beta_{Ap,v}$	-.788*
	$\beta_{Ap,vi}$	0.461
	$h_{Ap,i}$	0.3
	$h_{Ap,iii}$	0.382
	$h_{Ap,v}$	0.619
Am CLD fitting parameters	$\beta_{Am,1}$	0.463
	$\beta_{Am,2}$	-0.467
	$\beta_{Am,3}$	-0.551
	$h_{Am,1}$	-0.018
	$h_{Am,2}$	0.349
	$h_{Am,3}$	0.223
Branched SEC	$\overline{R}_{\mathrm{h}}$	-0.633
	$\overline{R}_{\mathrm{h,\ Am}}$	-0.515
	$\overline{R}_{\mathrm{h,\ Ap}}$	-0.647

[1] * Correlation is significant at the 0.05 level (two-tailed).

Table S3. Pearson correlations between rice sensory measured by panellists and relative contents of volatile compounds (n=8)[1].

	**Aroma intensity**	**Sulfurous**	**Eggy**	**Green vegetable**	**Root vegetables**	**Sweet caramel-ised**	**Brown bread**	**Earthy**	**Cereal / Porridge**	**Fragrant rice**	**Resinous**	**Plastic**	**Raw cookie dough**	**Card-board**	**Chemical**
cyclotetrasiloxane, octamethyl	-0.381	-0.205	-0.495	0.181	-0.101	-0.579	-0.07	0.29	0.183	-0.103	-0.221	0.462	-0.066	0.191	-0.043
undecane	-0.448	-0.053	0.136	0.421	-0.061	-0.347	0.216	0.136	0.28	-0.372	-0.156	-0.208	0.045	-0.102	-0.415
furfural	-0.518	-0.357	-0.63	0.308	0.145	-0.515	-0.239	0.354	0.266	-0.268	-0.367	0.46	-0.214	0.249	-0.032
hexanal	-0.657	-0.401	0.19	0.003	-0.263	-0.114	-0.174	-0.382	0.68	-0.029	-0.415	-0.27	-0.24	0.039	-0.253
3,7,11-trimethyl-1-dodecanol	-0.448	-0.151	0.473	-0.091	-0.346	-0.275	-0.249	-0.481	0.587	0.147	-0.248	-0.185	-0.25	0.085	-0.233
2-heptenal	0.1	-0.009	-0.294	0.503	0.467	-0.513	0.105	0.377	-0.213	-0.217	.725*	0.562	-0.499	0.111	0.219
dodecanal	-0.382	-0.346	-0.672	-0.142	-0.002	-0.082	-0.237	0.163	0.235	-0.037	-0.529	0.314	-0.011	0.35	0.054
dihydro-5-pentyl-2(3h)-furanone	-0.531	-.802*	-.842**	0.005	0.035	-0.184	-0.249	-0.026	0.421	0.067	-0.068	0.535	-0.603	0.229	0.367
dodecane	-0.191	-0.372	-0.165	0.054	-0.229	-0.372	0.08	-0.194	0.231	0.23	0.478	0.363	-0.497	-0.038	0.253
5-tridecene, (z)-	0.586	0.659	0.056	0.52	0.341	-0.069	0.243	.763*	-.820*	-0.426	0.039	-0.034	0.665	-0.429	-0.166
2-methoxy-phenol	-0.149	0.07	0.12	-0.349	-0.1	-0.249	-0.465	-0.248	0.296	0.241	-0.313	0.208	-0.156	0.511	-0.039
cis-vaccenic acid	0.634	0.459	0.419	0.319	0.347	0.416	0.214	0.24	-0.641	-0.232	0.3	-0.399	0.341	-0.555	-0.018
octadecane, 1-(ethenyloxy)-	-0.629	-0.237	-0.095	-0.067	-0.465	-0.234	0.098	-0.078	0.513	-0.065	-0.579	-0.154	0.206	0.098	-0.371
2,6,10-trimethyl- tetradecane	0.441	0.27	-0.452	0.172	0.22	0.064	0.214	0.618	-0.645	-0.175	0.062	0.241	0.45	-0.172	0.127
heptadecane	-0.454	-0.238	-0.205	0.627	0.371	-0.026	-0.144	0.405	0.142	-0.649	-0.609	-0.247	0.084	-0.248	-0.312
pentadecane	0.566	0.444	0.464	0.045	0.099	0.632	0.311	0.08	-0.53	-0.119	0.087	-0.608	0.557	-0.522	-0.11
4-methyldecane	-0.489	-0.568	-0.357	-0.161	-0.219	-0.445	-0.532	-0.296	0.544	0.311	-0.191	0.53	-0.601	0.294	0.262
cyclopentadecanone	-0.66	-0.332	-0.149	-0.139	-0.248	-0.352	-0.375	-0.188	0.631	0.037	-0.633	0.119	-0.17	0.377	-0.193
dodecamethyl-cyclohexasiloxane	-0.113	-0.118	-0.234	-0.222	-0.283	-0.231	-0.521	-0.022	0.106	0.257	-0.594	0.323	0.101	0.057	0.108
1-octanol	0.568	0.282	0.032	-0.003	-0.207	-0.193	0.262	0.12	-0.514	0.297	0.633	0.329	0.118	-0.309	0.294
2-tridecanone	-0.141	-0.058	0.048	-0.419	-0.533	-0.597	-0.245	-0.38	0.334	0.564	0.112	0.516	-0.301	0.381	0.149
nonanal	0.261	0.144	0.32	0.438	0.347	0.454	0.192	0.177	-0.339	-0.383	0.085	-0.543	0.212	-0.594	-0.114
tetradecane	-0.223	-0.064	0.273	0.38	0.054	0.311	0.114	0.079	0.077	-0.426	-0.444	-0.665	0.313	-0.511	-0.37
heptacosane	0.406	0.383	0.126	-0.223	-0.171	-0.597	-0.204	-0.038	-0.232	0.453	0.447	0.65	-0.157	0.285	0.251
benzene, 1,3-bis(1,1-dimethylethyl)	-.940**	-0.454	-0.028	-0.285	-0.368	-0.185	0.284	-0.466	.976**	-0.012	-0.16	-0.204	-0.334	0.648	-0.391
6-methyl-octadecane	.827*	0.634	0.166	0.064	0.239	0.087	0.147	0.346	-.796*	0.036	0.453	0.128	0.347	-0.202	0.165

1-octen-3-ol	-0.356	-0.489	-0.664	-0.043	0.343	-0.043	0.057	0.013	0.308	-0.077	0.327	0.404	-0.627	0.582	0.244
2-hexyl-1-octanol	-0.229	0.109	0.248	-0.142	-0.621	-0.337	0.15	-0.137	0.233	0.159	-0.279	-0.104	0.33	-0.059	-0.305
1-eicosanol	-0.686	-0.195	-0.178	0.089	-0.292	-0.457	0.127	0.071	0.516	-0.199	-0.446	0.008	0.077	0.269	-0.409
2-decen-1-ol	0.099	0.01	-0.288	0.674	0.311	-0.301	0.045	0.587	-0.372	-0.39	0.094	0.231	0.057	-0.431	0.045
2-decenal	-0.637	-0.308	-0.164	0.234	-0.195	-0.137	0.256	0.088	0.427	-0.328	-0.406	-0.254	0.125	-0.053	-0.382
2-methyl-1-hexadecanol	-0.411	0.068	0.249	-0.344	-0.701	-0.455	0.298	-0.319	0.492	0.253	-0.079	-0.031	0.128	0.344	-0.358
benzaldehyde	-0.155	-0.095	-0.32	-0.41	-0.307	-0.514	-0.164	-0.148	0.249	0.416	0.096	0.642	-0.272	0.59	0.187
8,8,9-trimethyl-deca-3,5-diene-2,7-dione	0.05	0.16	0.433	0.461	0.234	0.326	0.212	0.143	-0.153	-0.453	-0.104	-0.652	0.279	-0.528	-0.324
2-nonenal	-0.445	0.238	0.595	0.087	-0.262	-0.449	-0.055	-0.153	0.457	-0.158	-0.434	-0.314	0.144	0.189	-0.608
2-myristynoyl pantetheine	0.301	0.13	-0.275	0	0.034	-0.622	-0.509	0.206	-0.277	0.301	0.136	.846**	-0.222	0.151	0.391
nonadecane	-0.181	-0.19	-0.151	-0.003	0.118	0.649	-0.117	0.088	0.046	-0.287	-.793*	-0.535	0.41	-0.239	-0.179
10-methyl-e-11-tridecen-1-ol propionate	-0.116	0.037	0.1	-0.182	-0.573	-0.432	-0.14	-0.135	0.154	0.314	-0.263	0.191	0.167	-0.052	-0.061
2-nonen-1-ol	-0.538	-0.214	-0.212	-0.353	-0.381	-0.419	-0.221	-0.207	0.561	0.208	-0.436	0.278	-0.118	0.563	-0.133
oleic acid	0.447	0.213	-0.441	0.283	0.636	0.065	-0.049	0.59	-0.601	-0.269	0.215	0.353	0.049	0.034	0.243
dimethyl silanediol	-0.214	-0.027	-0.303	-0.043	-0.38	-0.449	0.298	0.158	0.099	0.065	0.014	0.297	0.156	0.148	-0.097
2-butyl-2-octenal	-0.061	-0.367	0.13	0.225	0.301	0.108	-0.56	-0.248	0.15	0.012	0.008	0	-0.576	-0.258	0.275
cyclooctasiloxane, hexadecamethyl	-0.254	0.21	0.166	0.315	0.068	-.782*	-0.462	0.205	0.171	-0.175	-0.381	0.304	-0.077	0.227	-0.274
geranyl isovalerate	-0.318	0.065	0.019	.721*	0.384	-0.68	-0.257	0.435	0.089	-0.523	-0.204	0.191	-0.175	0.008	-0.289
naphthalene	-0.338	-.853**	-0.677	-0.485	-0.406	0.126	-0.359	-0.438	0.428	0.553	-0.152	0.429	-0.477	0.06	0.567
z-8-methyl-9-tetradecenoic acid	0.082	0.484	0.418	0.074	0.017	0.246	.856**	0.133	-0.131	-0.355	0.285	-0.625	0.495	0.046	-0.529
methoxy-phenyl-oxime	-.751*	-0.386	-0.078	0.184	-0.003	-0.511	-0.244	-0.132	0.687	-0.173	-0.274	0.129	-0.475	0.374	-0.227
1,2-15,16-diepoxyhexadecane	-.909**	-0.169	0.073	-0.26	-0.41	-0.204	0.386	-0.273	.867**	-0.164	-0.442	-0.365	0.077	0.66	-0.661
10-heptadecen-8-ynoic acid, methyl ester, (e)-	-0.019	0.082	-0.019	-0.076	-0.291	-.782*	-0.096	-0.062	0.098	0.333	0.362	0.637	-0.301	0.291	0.132
cis-7-hexadecenoic acid	-0.393	-0.125	0.491	-0.394	-0.487	0.423	0.006	-0.557	0.529	0.118	-0.62	-.737*	0.269	-0.053	-0.382
2-hexadecanol	-0.414	-0.364	-0.032	-0.345	-0.604	0.329	0.698	-0.398	0.444	0.146	0.095	-0.445	0.13	-0.041	-0.174
cholestan-3-ol, 2-methylene-, (3β,5α)-	-0.297	-0.617	-0.441	-0.253	-0.345	-0.203	0.142	-0.365	0.389	0.418	0.529	0.456	-0.624	0.175	0.413
2h-pyran, tetrahydro-2-(12-	-0.363	-0.156	-0.543	0.191	0.247	0.164	-0.004	0.463	0.056	-0.489	-.736*	-0.127	0.364	0.103	-0.262

pentadecynyloxy)-															
2-methyl-e,e-3,13-octadecadien-1-ol	-0.403	-0.109	0.268	-0.439	-.831*	0.041	0.322	-0.448	0.485	0.276	-0.355	-0.409	0.349	-0.022	-0.337
2,4-di-tert-butylphenol	-0.218	0.107	0.069	-0.044	-0.463	-0.086	0.099	0.098	0.094	-0.058	-0.657	-0.26	0.612	-0.194	-0.375
hexadecanoic acid, methyl ester	0.294	0.542	0.67	0.182	-0.259	-0.031	0.083	0.099	-0.305	-0.082	-0.27	-0.446	0.648	-0.545	-0.362
9,11-octadecadienoic acid, methyl ester, (e,e)-	0.116	0.151	-0.255	.818*	.812*	-0.608	-0.586	.707*	-0.361	-0.511	-0.065	0.562	-0.286	-0.034	0.087
(e)-2-dodecenal	-0.225	-0.13	-0.215	-0.423	-0.391	-0.35	-0.368	-0.195	0.302	0.385	-0.358	0.412	-0.071	0.393	0.088
tert-hexadecanethiol	-0.551	-0.185	0.246	-0.059	-0.222	-0.326	-0.436	-0.311	0.603	0.034	-0.559	-0.074	-0.192	0.222	-0.263
hexadecane	0.369	0.183	0.126	-.737*	-0.681	0.103	0.206	-0.4	-0.112	.709*	0.273	0.129	0.238	0.074	0.229
6,10-dimethyl-5,9-undecadien-2-one	-0.257	0.407	0.18	0.031	-0.047	-0.616	-0.185	0.201	0.188	-0.173	-0.48	0.13	0.234	0.508	-0.474
estra-1,3,5(10)-trien-17β-ol	-0.162	0.015	0.405	-0.431	-0.445	0.552	-0.027	-0.399	0.266	0.128	-.721*	-.711*	0.507	-0.153	-0.32

[1] * Correlation is significant at the 0.05 level (two-tailed); ** Correlation is significant at the 0.01 level (two-tailed).

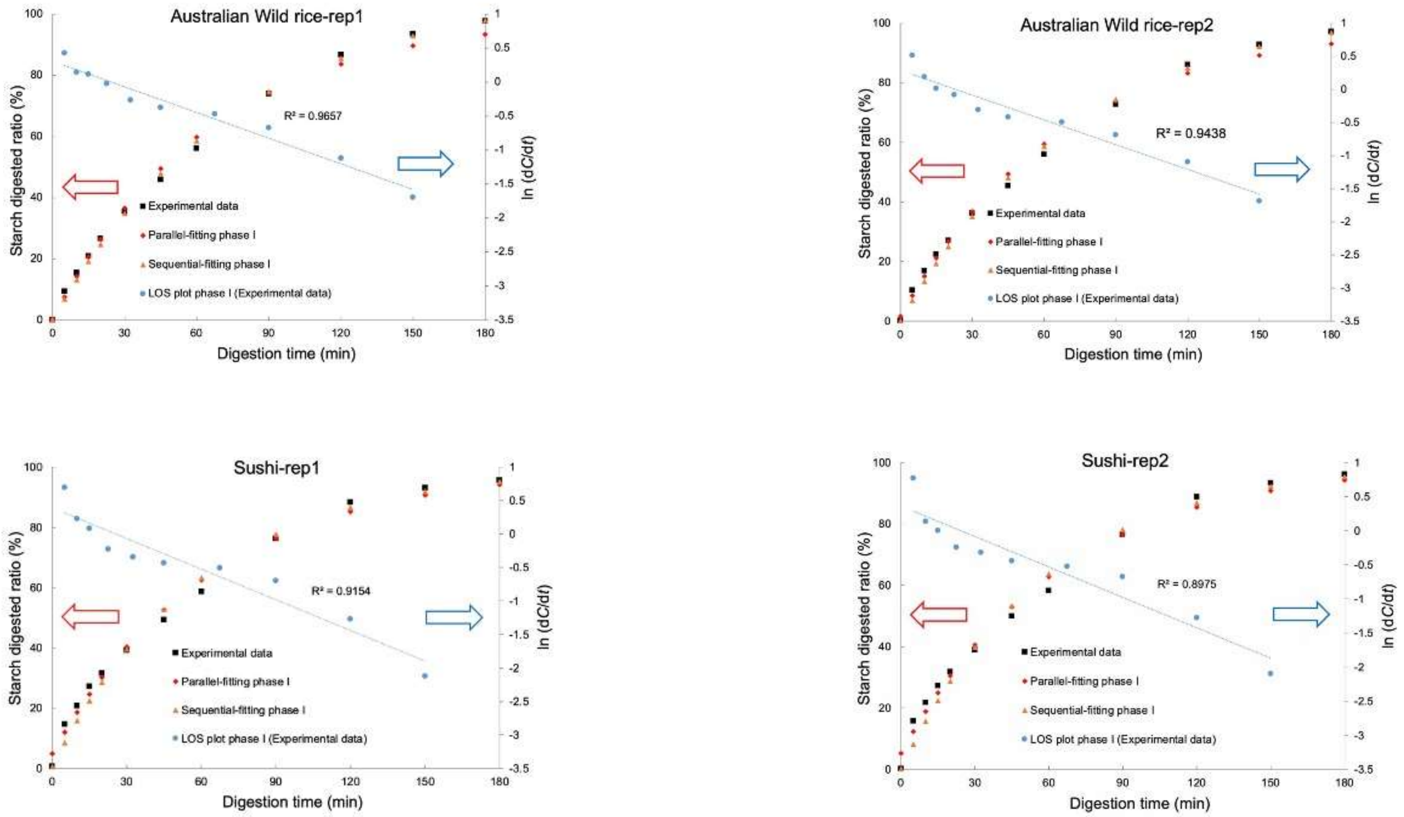

Australian Wild rice-rep1
R² = 0.9657
Experimental data
Parallel-fitting phase I
Sequential-fitting phase I
LOS plot phase I (Experimental data)
Starch digested ratio (%)
ln (dC/dt)
Digestion time (min)
Australian Wild rice-rep2
R² = 0.9438
Experimental data
Parallel-fitting phase I
Sequential-fitting phase I
LOS plot phase I (Experimental data)
Starch digested ratio (%)
ln (dC/dt)
Digestion time (min)
Sushi-rep1
R² = 0.9154
Experimental data
Parallel-fitting phase I
Sequential-fitting phase I
LOS plot phase I (Experimental data)
Starch digested ratio (%)
ln (dC/dt)
Digestion time (min)
Sushi-rep2
R² = 0.8975
Experimental data
Parallel-fitting phase I
Sequential-fitting phase I
LOS plot phase I (Experimental data)
Starch digested ratio (%)
ln (dC/dt)
Digestion time (min)

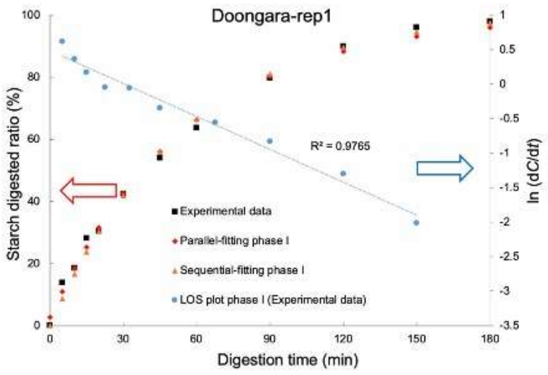
Doongara-rep1
Starch digested ratio (%)
ln (dC/dt)
Digestion time (min)
R² = 0.9765
Experimental data
Parallel-fitting phase I
Sequential-fitting phase I
LOS plot phase I (Experimental data)

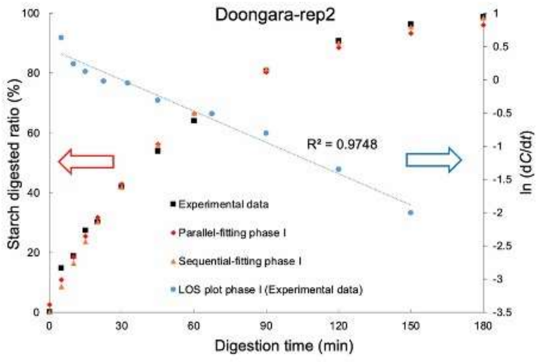
Doongara-rep2
Starch digested ratio (%)
ln (dC/dt)
Digestion time (min)
R² = 0.9748
Experimental data
Parallel-fitting phase I
Sequential-fitting phase I
LOS plot phase I (Experimental data)

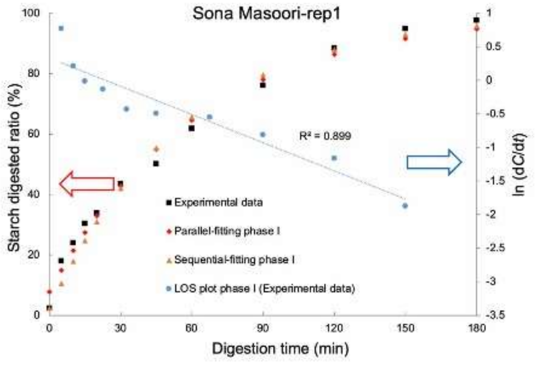
Sona Masoori-rep1
Starch digested ratio (%)
ln (dC/dt)
Digestion time (min)
R² = 0.899
Experimental data
Parallel-fitting phase I
Sequential-fitting phase I
LOS plot phase I (Experimental data)

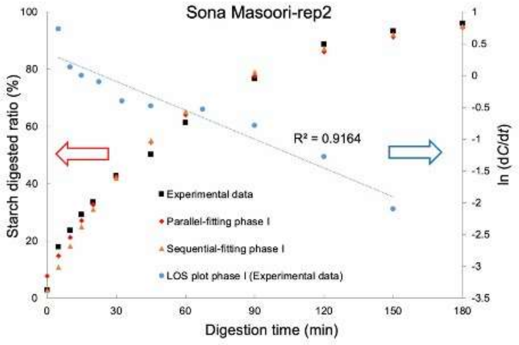
Sona Masoori-rep2
Starch digested ratio (%)
ln (dC/dt)
Digestion time (min)
R² = 0.9164
Experimental data
Parallel-fitting phase I
Sequential-fitting phase I
LOS plot phase I (Experimental data)

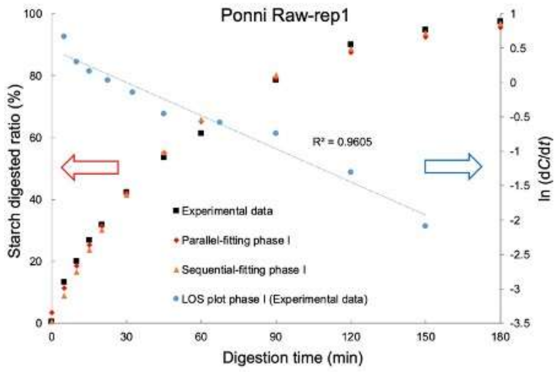
Ponni Raw-rep1
R² = 0.9605
Experimental data
Parallel-fitting phase I
Sequential-fitting phase I
LOS plot phase I (Experimental data)
Starch digested ratio (%)
ln (dC/dt)
Digestion time (min)

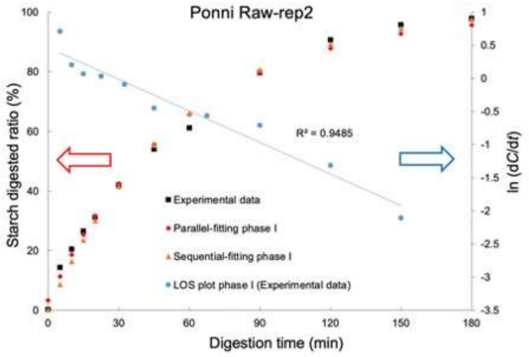
Ponni Raw-rep2
R² = 0.9485
Experimental data
Parallel-fitting phase I
Sequential-fitting phase I
LOS plot phase I (Experimental data)
Starch digested ratio (%)
ln (dC/dt)
Digestion time (min)

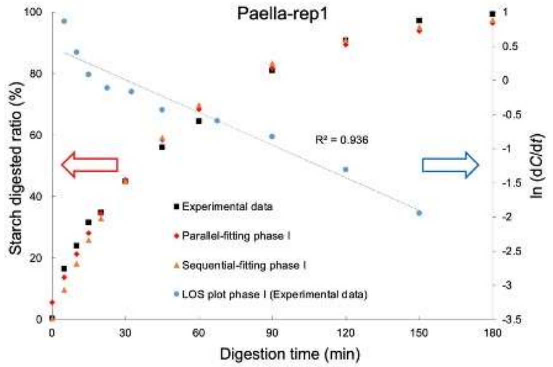
Paella-rep1
R² = 0.936
Experimental data
Parallel-fitting phase I
Sequential-fitting phase I
LOS plot phase I (Experimental data)
Starch digested ratio (%)
ln (dC/dt)
Digestion time (min)

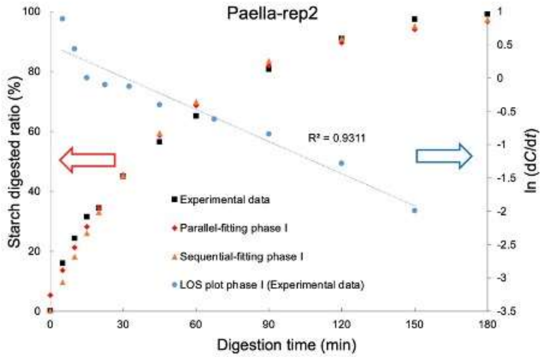
Paella-rep2
R² = 0.9311
Experimental data
Parallel-fitting phase I
Sequential-fitting phase I
LOS plot phase I (Experimental data)
Starch digested ratio (%)
ln (dC/dt)
Digestion time (min)

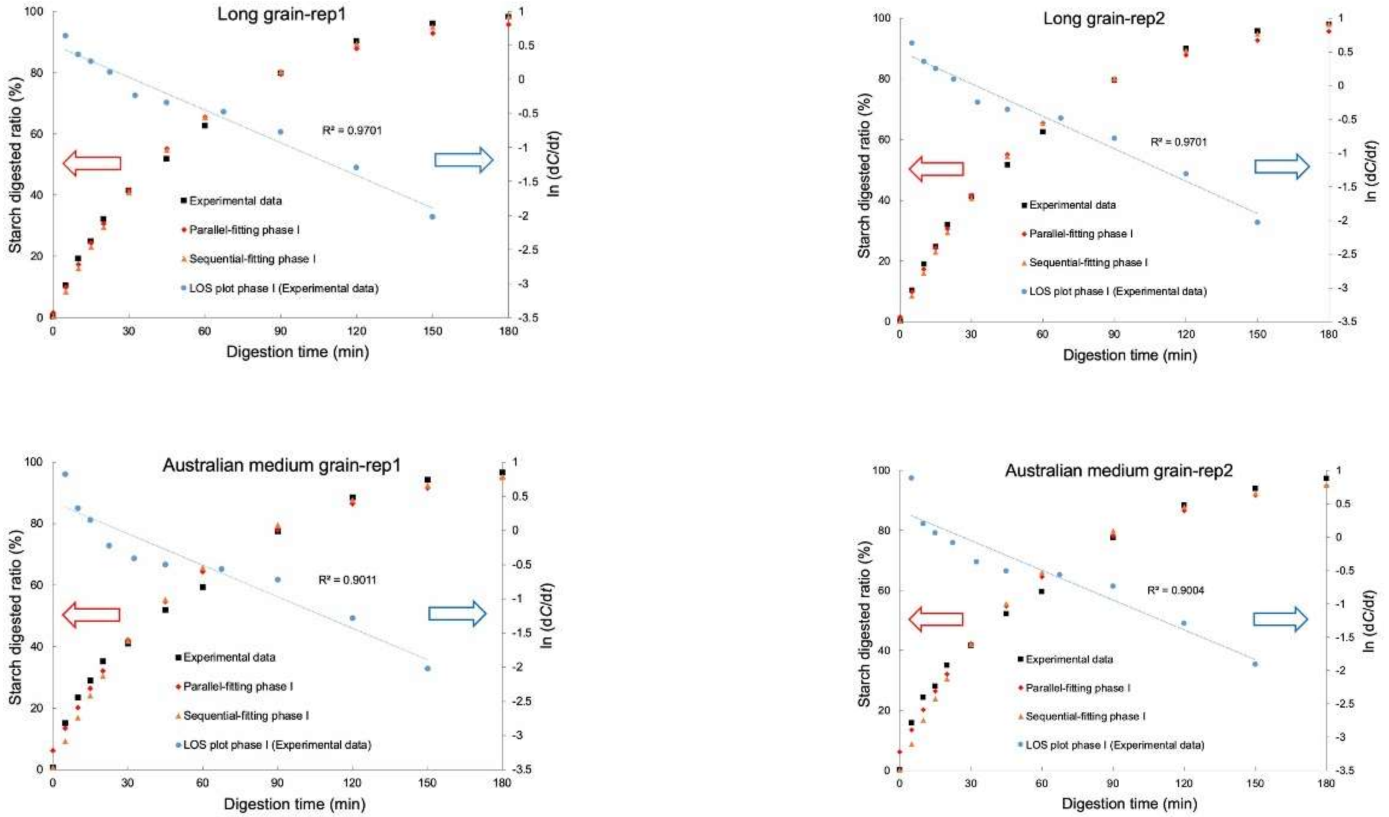

Figure S1. Typical starch digestion curves, model-fit (Parallel-fitting and Sequential-fitting) curves and LOS plots for cooked AWR compared to CRs (Sushi, Doongara, Sona Masoori, Ponni Raw, Paella, Long grain, and Australian Medium grain). Rep1 and rep2 are two replicates.

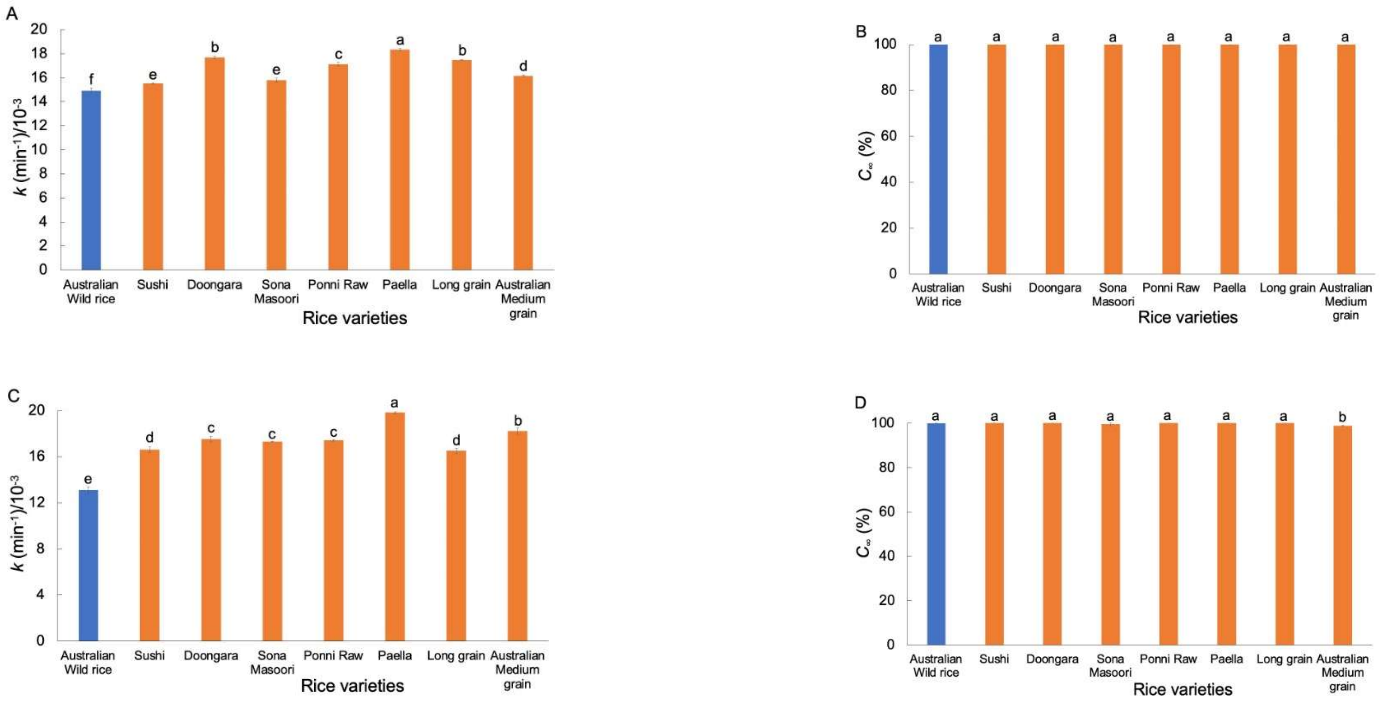
A
k (min-1)/10-3
Rice varieties
Australian Wild rice
Sushi
Doongara
Sona Masoori
Ponni Raw
Paella
Long grain
Australian Medium grain
B
C∞ (%)
C
D

Figure S2. Comparison of in vitro digestibility parameters of AWR and CRs. (A-B) The parameters from Parallel-fitting model; (C-D) The parameters from Sequential-fitting model. Blue and orange: AWR and CRs (Sushi, Doongara, Sona Masoori, Ponni Raw, Paella, Long grain, and Australian Medium grain), respectively. All data were from duplicate measurements. The same letters mean not significant difference ($p < 0.05$). k is the digestion rate coefficient of starch and C_∞ is the percentage of starch digested by the end of reaction time.

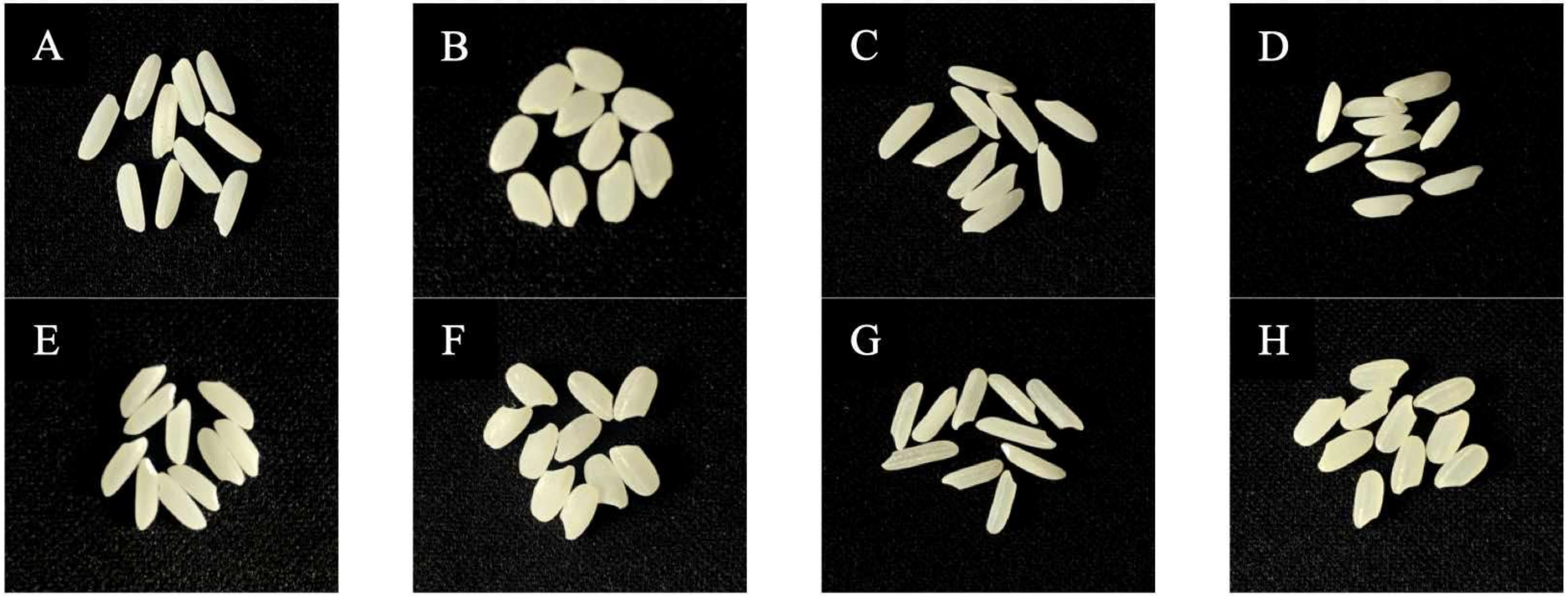

Figure S3. (A-H) The visual pictures of AWR and CRs (Sushi, Doongara, Sona Masoori, Ponni Raw, Paella, Long grain, and Australian Medium grain), respectively.

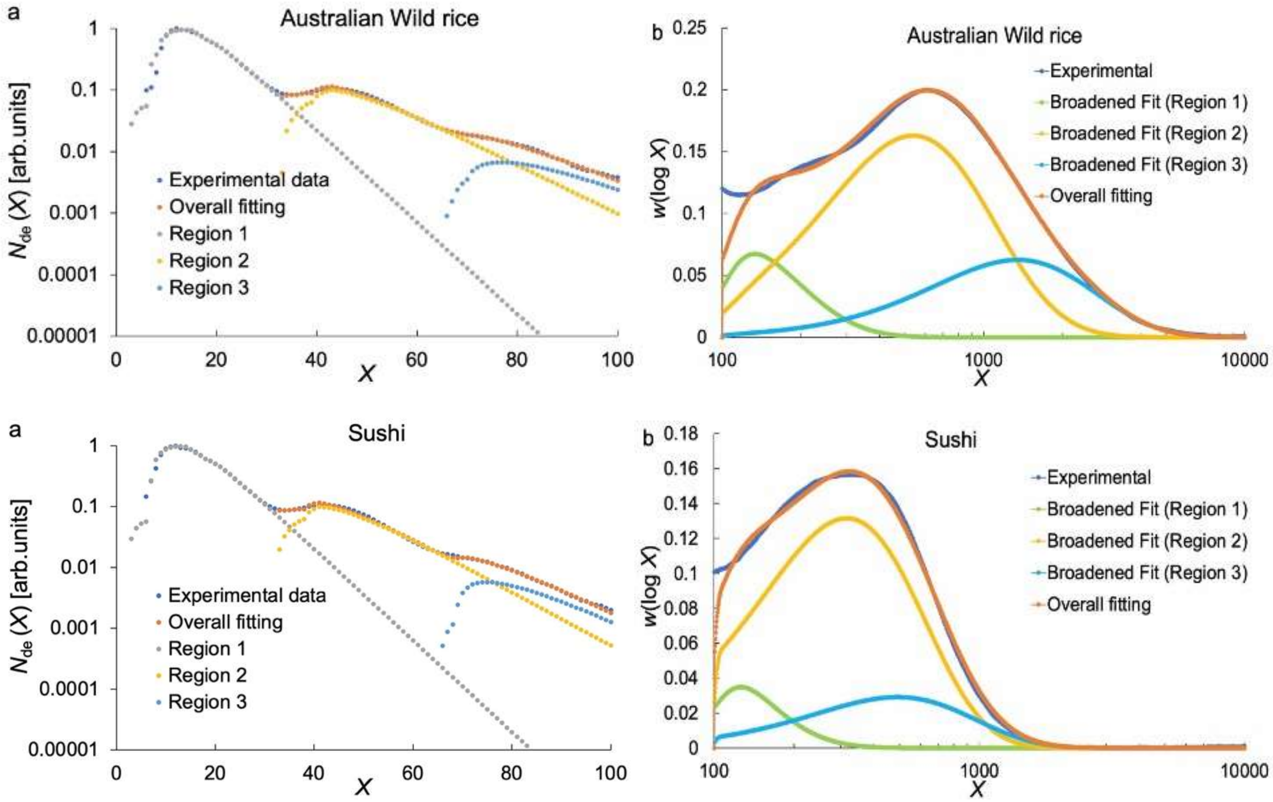
a
Australian Wild rice
$N_{de}(X)$ [arb.units]
Experimental data
Overall fitting
Region 1
Region 2
Region 3
X
b
Australian Wild rice
w(log X)
Experimental
Broadened Fit (Region 1)
Broadened Fit (Region 2)
Broadened Fit (Region 3)
Overall fitting
X
a
Sushi
$N_{de}(X)$ [arb.units]
Experimental data
Overall fitting
Region 1
Region 2
Region 3
X
b
Sushi
w(log X)
Experimental
Broadened Fit (Region 1)
Broadened Fit (Region 2)
Broadened Fit (Region 3)
Overall fitting
X

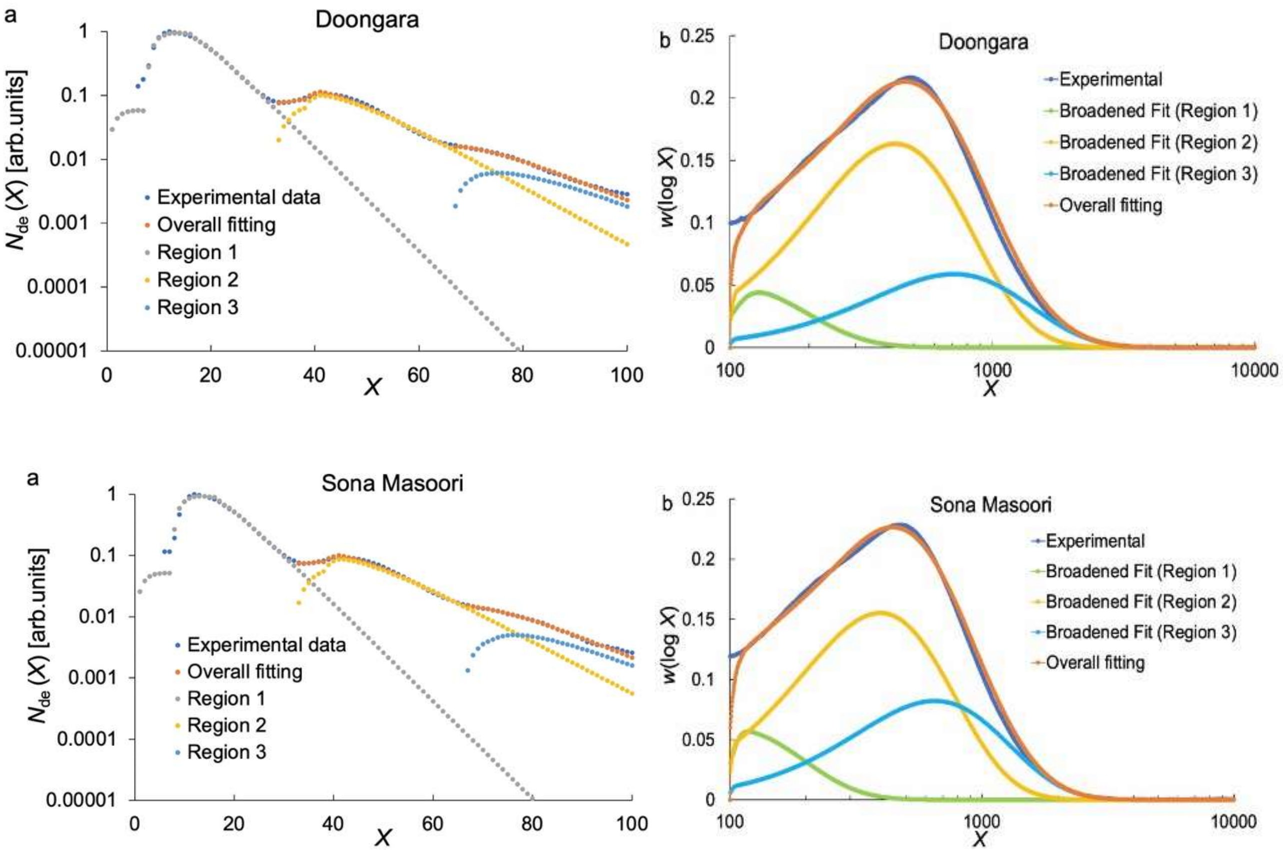
a
Doongara
$N_{de}(X)$ [arb.units]
X
Experimental data
Overall fitting
Region 1
Region 2
Region 3
b
Doongara
w(log X)
X
Experimental
Broadened Fit (Region 1)
Broadened Fit (Region 2)
Broadened Fit (Region 3)
Overall fitting
a
Sona Masoori
$N_{de}(X)$ [arb.units]
X
Experimental data
Overall fitting
Region 1
Region 2
Region 3
b
Sona Masoori
w(log X)
X
Experimental
Broadened Fit (Region 1)
Broadened Fit (Region 2)
Broadened Fit (Region 3)
Overall fitting

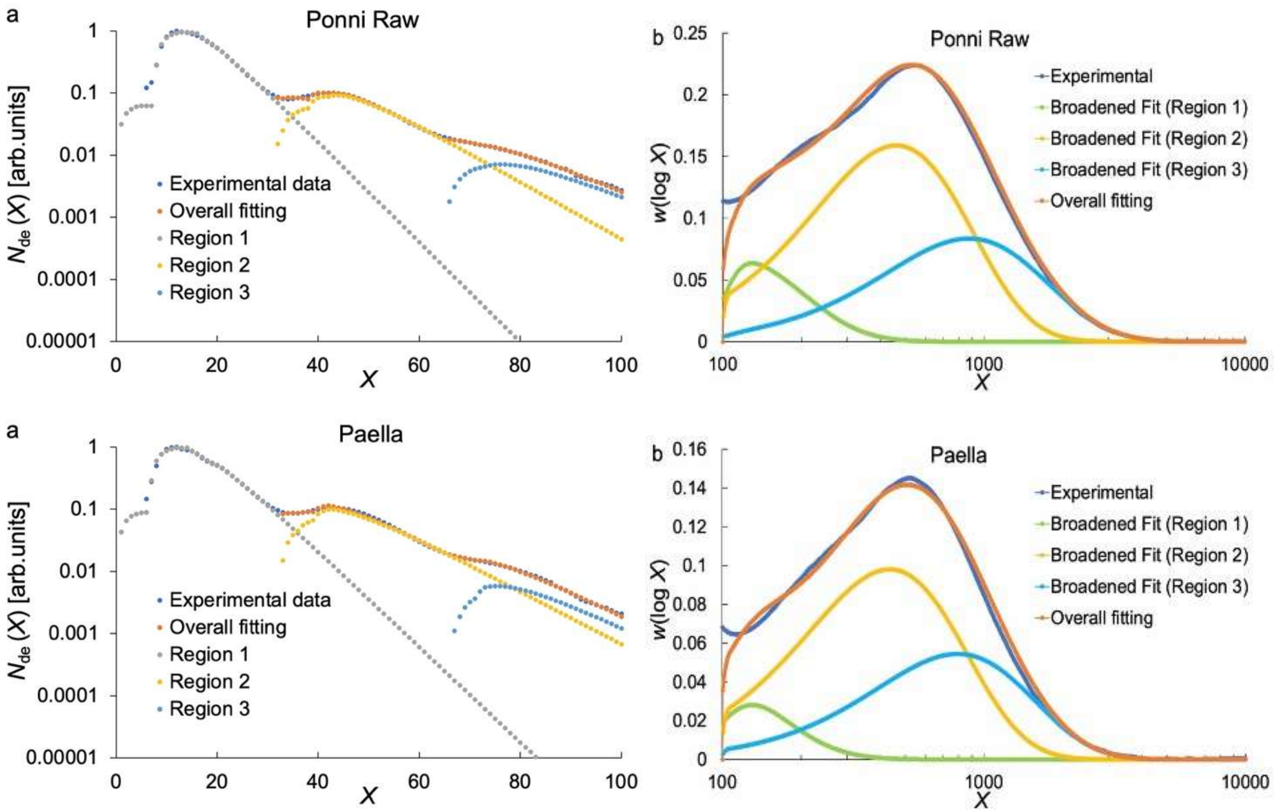

a
Ponni Raw
1
0.1
0.01
0.001
0.0001
0.00001
$N_{de}(X)$ [arb.units]
0
20
40
60
80
100
X
Experimental data
Overall fitting
Region 1
Region 2
Region 3
b
Ponni Raw
0.25
0.2
0.15
0.1
0.05
0
w(log X)
100
1000
10000
X
Experimental
Broadened Fit (Region 1)
Broadened Fit (Region 2)
Broadened Fit (Region 3)
Overall fitting
a
Paella
1
0.1
0.01
0.001
0.0001
0.00001
$N_{de}(X)$ [arb.units]
0
20
40
60
80
100
X
Experimental data
Overall fitting
Region 1
Region 2
Region 3
b
Paella
0.16
0.14
0.12
0.1
0.08
0.06
0.04
0.02
0
w(log X)
100
1000
10000
X
Experimental
Broadened Fit (Region 1)
Broadened Fit (Region 2)
Broadened Fit (Region 3)
Overall fitting

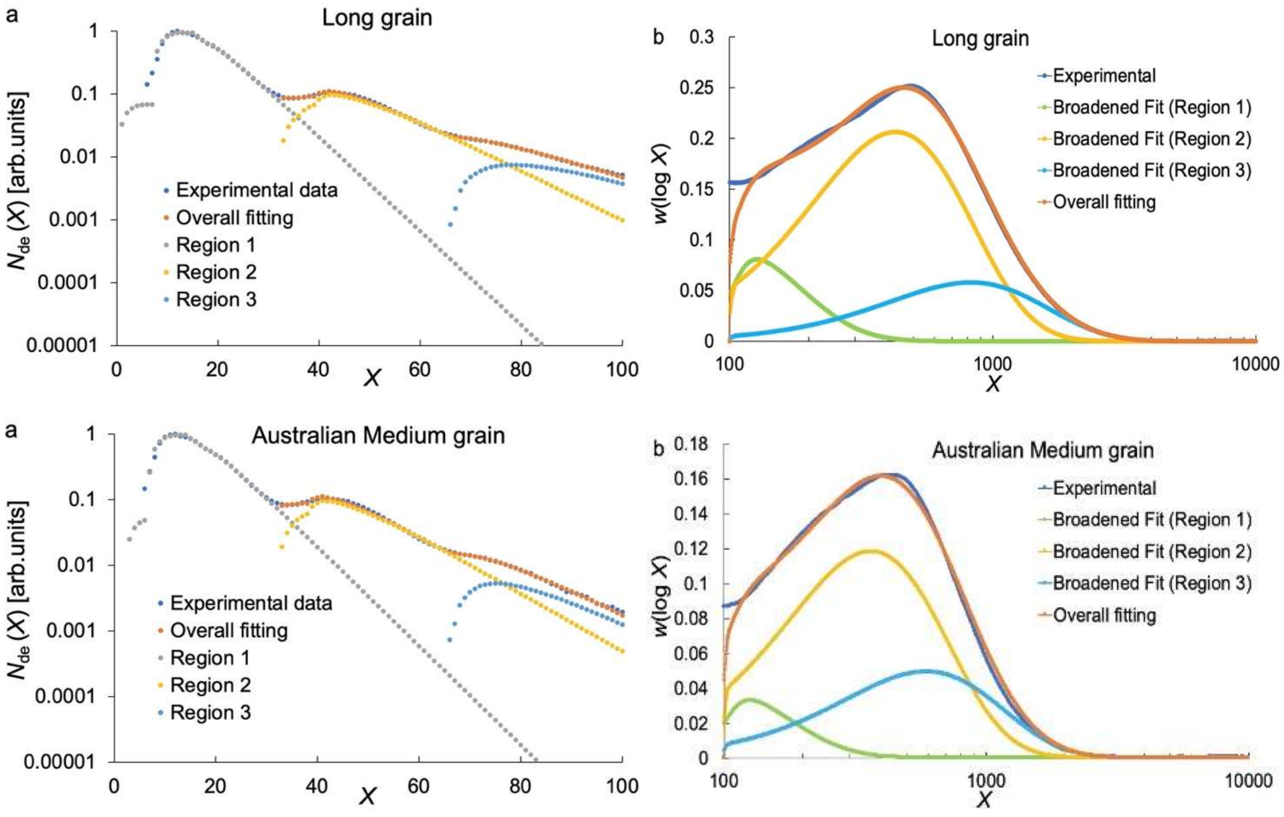

Figure S4. Fitting results of Ap (a) and Am (b) CLD for AWR and CRs (Sushi, Doongara, Sona Masoori, Ponni Raw, Paella, Long grain, and Australian Medium grain).
